# ANLN-induced EZH2 upregulation promotes pancreatic cancer progression by mediating miR-218-5p/LASP1 signaling axis

**DOI:** 10.1186/s13046-019-1340-7

**Published:** 2019-08-08

**Authors:** Anbin Wang, Haisu Dai, Yi Gong, Chengcheng Zhang, Junjie Shu, Yuandeng Luo, Yan Jiang, Wei Liu, Ping Bie

**Affiliations:** Department of Hepatobiliary Surgery, Southwest Hospital, Third Military Medical University (Army Medical University), Chongqing, China

**Keywords:** Pancreatic cancer, ANLN, EZH2, miR-218-5p, LASP1

## Abstract

**Background:**

Pancreatic cancer is a highly lethal malignancy with poor prognosis. Anillin (ANLN), an actin binding protein, is upregulated and plays an important role in many malignant tumors. However, the precise role of ANLN in pancreatic cancer remains unclear.

**Methods:**

The expression of ANLN and its association with pancreatic cancer patient survival were analyzed using an online database and confirmed by immunohistochemistry. The ANLN protein expression in pancreatic cancer cell lines was detected by Western blot. Cell proliferation, colony formation and transwell assays in vitro and in vivo tumor growth were used to determine the role of ANLN in pancreatic cancer. Gene expression microarray analysis and a series of in vitro assays were used to elucidate the mechanisms of ANLN regulating pancreatic cancer progression.

**Results:**

We found that the ANLN expression was significantly upregulated in pancreatic cancer tissues and cell lines. The high expression of ANLN was associated with tumor size, tumor differentiation, TNM stage, lymph node metastasis, distant metastasis and poor prognosis in pancreatic cancer. ANLN downregulation significantly inhibited cell proliferation, colony formation, migration, invasion and tumorigenicity in nude mice. Meanwhile, we found that ANLN knockdown inhibited several cell-cell adhesion related genes, including the gene encoding LIM and SH3 protein 1 (LASP1). LASP1 upregulation partially reversed the tumor-suppressive effect of ANLN downregulation on pancreatic cancer cell progression. Moreover, we found that ANLN downregulation induced the expression of miR-218-5p which inhibited LASP1 expression through binding to its 3’UTR. We also found that ANLN-induced enhancer of zeste homolog 2 (EZH2) upregulation was involved in regulating miR-218-5p/LASP1 signaling axis. EZH2 upregulation or miR-218-5p downregulation partially reversed the tumor-suppressive effect of ANLN downregulation on pancreatic cancer cell progression.

**Conclusion:**

ANLN contributed to pancreatic cancer progression by regulating EZH2/miR-218-5p/LASP1 signaling axis. These findings suggest that ANLN may be a candidate therapeutic target in pancreatic cancer.

**Electronic supplementary material:**

The online version of this article (10.1186/s13046-019-1340-7) contains supplementary material, which is available to authorized users.

## Background

Pancreatic cancer is a fatal malignancy with a poor prognosis worldwide [1]. Due to occult onset and non-specific symptoms, 80% of patients diagnosed with pancreatic cancer are in advanced stages and have a 5-year survival rate of less than 5% [2, 3]. Despite ongoing advances for the survival rates noted in many cancers, such as colon cancer and breast cancer, the annual mortality rates for patients with pancreatic cancer remain almost equal to the incidence rates [3, 4]. Thus, to improve the health outcomes of pancreatic cancer patients, more intensive efforts should be made to understand the molecular mechanisms underlying pancreatic cancer progression.

Anillin (ANLN), an actin binding protein, first identified in *Drosophila*, is located on chromosome 7p14.2 and encodes an actin-binding protein that consists of 1125 amino acids and plays an important role in cytokinesis [5–7]. In normal tissues, ANLN expression is higher in the placenta, brain and testis, and lower in the lung, heart, liver and spleen [8]. Many recent studies suggests that ANLN is upregulated in numerous cancer types, including cervical cancer, prostate cancer, anaplastic thyroid carcinoma, breast cancer, lung carcinogenesis, bladder urothelial carcinoma, pancreatic cancer and nasopharyngeal carcinoma [9–16]. Functionally, increasing evidence has indicated that ANLN is critical for growth and metastasis of cancer cells. For example, ANLN knockdown inhibited cell proliferation and metastasis and induced G2/M arrest in bladder urothelial carcinoma [16]. In non-small cell lung cancer cells, ANLN downregulation induced cell growth repression, and ANLN overexpression promoted cell motility [17]. Moreover, ANLN was upregulated in pancreatic cancer and was involved in miR-217-mediated cell proliferation and invasion [18]. Nevertheless, the mechanisms that underlie the role of ANLN in the regulation of pancreatic cancer progression have not been fully addressed.

LIM and SH3 protein 1 (LASP1), a structural scaffolding protein and adhesion adaptor protein, was initially identified in breast cancer and was located at 17q12 [19]. Previous studies showed that LASP1 was upregulated in many malignant tumors including nasopharyngeal carcinoma, breast cancer, glioblastoma and colorectal cancer and contributed to tumor proliferation, invasion and metastasis [20–23]. In addition, LASP1 is upregulated in pancreatic ductal adenocarcinoma and is essential for HIF1α-induced invasion and metastasis [24]. Moreover, our gene microarray analysis showed that ANLN downregulation repressed LASP1 expression. Whether LASP1 is involved in ANLN-induced pancreatic cancer progression is what we deal with in this study.

Recently, miRNAs have been shown to be deregulated in pancreatic cancer, affecting several steps of initiation and aggressiveness of the disease by directly regulating target genes expression [25]. Among these miRNAs, miR-218-5p was found to play pivotal roles in many malignant tumors [26–28]. For example, miR-218-5p upregulation repressed gastric cancer growth and metastasis by directly regulating CDK6/CyclinD1 [28]. Additionally, miR-218 was downregulated in pancreatic cancer tissues when compared with adjacent normal tissues, and reduced miR-218 was associated with poor prognosis of pancreatic cancer patients [29]. MiR-218 upregulation inhibited the proliferation and invasion and induced apoptosis of pancreatic cancer cells [30]. Interestingly, miR-218 could suppress cell proliferation, migration and invasion in gastric cancer and prostate cancer [31, 32]. Moreover, our gene microarray analysis showed that ANLN downregulation induced miR-218 expression. Thus, it is necessary to study the roles of miR-218-5p in ANLN-induced pancreatic cancer progression.

Enhancer of zeste homolog 2 (EZH2), a histone methyltransferase, is highly amplified in human cancers and plays an important role in the development and progression of cancers [33, 34]. In 2011, Cao Q et al. first found that EZH2 contributed to miRNAs dysregulation via the PRC2/PRC1 axis [35]. In pancreatic cancer, EZH2 downregulation induced the expression of miR-139-5p via H3K27me3, thereby repressing the progression of pancreatic cancer [36]. In addition, EZH2-mediated formation of heterochromatin silenced miR-218 in human pancreatic ductal adenocarcinoma cells [37]. Our gene microarray analysis showed that ANLN downregulation inhibited EZH2 expression. However, the relationship between EZH2/miR-218 axis and ANLN in pancreatic cancer progression has not been studied before.

In this study, we showed that ANLN expression was upregulated in pancreatic cancer tissues and cell lines. A high expression level of ANLN was associated with poor prognosis of pancreatic cancer patients. ANLN downregulation inhibited pancreatic cancer cell proliferation, colony formation, migration and invasion. In addition, EZH2/miR-218-5p/LASP1 signaling axis might be involved in ANLN-mediated cell proliferation, colony formation, migration and invasion in pancreatic cancer.

## Materials and methods

### Cell lines and cell culture

Five pancreatic cancer cell lines were acquired from the Cell Bank Type Culture Collection of the Chinese Academy of Sciences (Shanghai, China). The human pancreatic duct epithelial cell line (hTERT-HPNE) was obtained from the American Type Culture Collection (Manassas, VA, USA). The AsPC-1 and BxPC-3 cells were cultured in RPMI 1640 medium (Gibco, Grand Island, NY, USA) supplemented with 10% fetal bovine serum (Gibco, USA). The PANC-1 and MIA PaCa-2 cells were grown in DMEM (Gibco, USA) containing 10% fetal bovine serum. The SW1990 cell line was maintained in L-15 medium (Gibco, USA) supplemented with 10% fetal bovine serum. The hTERT-HPNE cell line was cultured in ATCC-suggested complete growth medium (1 V Medium M3, 3 V glucose-free DMEM, 5% fetal bovine serum, 10 ng/ml EGF, 5.5 mM D-glucose and 750 ng/ml puromycin).

### Patients and cancer tissues

This study was approved by the Ethical Committee of Army Medical University, China. All patients provided written informed consent to participate in the study. The study methodologies conformed to the standards set by the Declaration of Helsinki. Eighty pancreatic cancer tissues and ten cancer-adjacent normal tissues were obtained from the Southwest Hospital, Army Medical University (Chongqing, China) between December 2009 and June 2011. All tumor tissues were diagnosed as pancreatic cancer independently by two pathologists. Patients who had received chemotherapy or radiotherapy were excluded. The overall survival (OS) was defined as the interval between the date of definite diagnosis and the date of death or the last follow up. Collection of follow-up data was ceased in July 2016.

### Immunohistochemistry (IHC) analysis

Tissue sections were dewaxed in xylene for 20 min at 37 °C and then rehydrated with a series of graded alcohols. To eliminate endogenous peroxidase activity, the sections were incubated with endogenous peroxidase blocking buffer (Beyotime, Shanghai, China). The sections were subsequently treated with a citrate antigen retrieval solution (Beyotime, China) for 20 min at 98 °C. After washing, the sections were blocked with normal goat serum (Boster, Wuhan, China) for 20 min at room temperature. The sections were incubated with a mouse monoclonal antibody against human ANLN (1200; Abcam, Cambridge, United Kingdom) overnight at 4 °C. The sections were then washed and incubated with goat anti-mouse secondary antibodies (Boster, Wuhan, China) for 30 min at room temperature. Finally, the expression levels of ANLN were analyzed according to methods reported [38]. The degree of immunostaining of the sections was defined by the sum of a proportion score and an intensity score. The proportion score was defined as follows: 0, no positive immunoreactive cells; 1, ≤10% positive immunoreactive cells; 2, 10 to 50% positive immunoreactive cells; or 3, > 50% positive immunoreactive cells. The intensity score was defined as follows: 0, no immunoreactive staining; 1, weak immunoreactive staining; 2, intermediate immunoreactive staining; or 3, strong immunoreactive staining. The scores were independently evaluated by 2 pathologists.

### Small interfering RNA (siRNA), miRNA mimic, miRNA inhibitor, vector transfection and lentiviral particle infection

SiRNAs against ANLN and EZH2 were designed and synthesized by Sesh-biotech (Shanghai, China) (Additional file 1: Table S1). The pCMV3-LASP1 CDS (NM_006148) and pCMV3-EZH2 CDS (NM_004456) expression plasmids were acquired from Sino Biological Inc. (Beijing, China). Mimic control (con), miR-218-5p mimics (miR-218-5p), inhibitor control (anti-con) and miR-218-5p inhibitors (anti-miR-218) were obtained from GenePharma (Shanghai, China). For transfection, BxPC-3 and SW1990 cells were cultured in 6-well plates. When the BxPC-3 and SW1990 cells were 80% confluent, they were transfected with the negative control siRNA (NC), ANLN siRNA (ANLN RNAi) or EZH2 siRNA (EZH2 RNAi) using Lipofectamine 2000 (Invitrogen) according to the manufacturer’s instructions. For the rescue experiments, ANLN siRNA together with the pCMV3-LASP1 expression plasmid (LASP1) or pCMV3-EZH2 expression plasmid (EZH2), or miR-218-5p mimic together with the pCMV3-LASP1 expression plasmid (LASP1), or EZH2 siRNA together with the miR-218-5p inhibitor were transfected to BxPC-3 and SW1990 cells. To establish the stable ANLN-silencing BxPC-3 cell line, short hairpin RNA (shRNA) oligonucleotide sequences that targeted ANLN were cloned into the pLV-hU6-shRNA-CMV-puromycin lentiviral vector by Sesh-biotech (Shanghai, China). Lentiviral Packaging System was then used for lentivirus packaging. The shRNA sequences are listed in Additional file 1: Table S1. BxPC-3 cells were infected with lentivirus at an MOI (multiplicity of infection) = 15 and selected with 3 μg/ml puromycin for 15 days.

### Western blot analysis

Cells were harvested, and the total cellular proteins were extracted using RIPA buffer (Sigma-Aldrich, St. Louis, MO, USA) that contained a protease inhibitor cocktail (Roche, Basel, Switzerland). A BCA protein assay kit (Beyotime, China) was subsequently used to quantify the protein concentration. The proteins were then denatured in SDS sample buffers for 10 min at 100 °C, separated via SDS-PAGE and blotted onto PVDF membranes (Bio-Rad Laboratories, Hercules, CA, USA). The membranes were blocked in Tris-buffered saline that contained 5% nonfat powdered milk for 1 h. The membranes were subsequently probed with a mouse monoclonal antibody against human ANLN (1:500; Abcam, United Kingdom), mouse monoclonal antibody against human β-actin (1:3000; Proteintech, Wuhan, China), rabbit polyclonal antibody against human LASP1 (1:3000; Proteintech, China) or rabbit polyclonal antibody against human EZH2 (1:2000; Proteintech, China) at 4 °C overnight. After washing, the membranes were incubated with goat anti-mouse/rabbit secondary antibodies (1:3000; Santa Cruz Biotechnology, Dallas, TX, USA) at room temperature for 2 h. Finally, the ECL system (Thermo Scientific, Rockford, IL, USA) was used to visualize the protein band signals. The band density was analyzed by Quantity One v4.6.2 (Bio-Rad Laboratories, USA).

### Cell proliferation assay

For the cell proliferation assay, transfected BxPC-3 and SW1990 cells were seeded onto 96-well plates at a final concentration of 2000 cells/well and further incubated for 0, 1, 2, 3, and 4 d at 37 °C. Following incubation, the cell viability was measured using a Cell Counting Kit-8 (CCK-8) kit (Beyotime, China) according to the manufacturer’s instructions. The CCK-8 assays were repeated 3 times.

### Colony formation assay

After 48 h of transfection, BxPC-3 and SW1990 cells were collected, seeded onto 6-well plates at a final concentration of 500 cells/well and cultured for an additional 14 days. The cells were then stained with 0.05% crystal violet for 20 min. The number of colonies was evaluated via light microscopy. The colony formation assays were performed 3 times.

### Cell migration and invasion assay

The cell migration assay was performed using transwell chambers (BD Biosciences, USA) with a pore size of 8 μm. The cell invasion assay was performed using Matrigel-coated transwell chambers (BD Biosciences, USA) with a pore size of 8 μm. At 48 h after transfection, BxPC-3 and SW1990 cells were seeded onto the upper chambers at a final concentration of 5 × 10^4^ cells/well and cultured in 100 μl of serum-free medium. The lower chambers contained 700 μl of medium with 10% FBS. After 48 h of incubation, the cells migrated or invaded through the filter into the lower side of the chamber were fixed and stained with crystal violet for 30 min. The number of cells was counted under a microscope. Each experiment was performed in triplicate.

### In vivo xenograft tumor models

This study was approved by the Ethical Committee of Army Medical University, China. The stable ANLN-silenced BxPC-3 cells (LV-ANLN shRNA, 1 × 10^6^ cells in 100 μl of sterilized PBS) and the stable scramble control BxPC-3 cells (LV-NC, 1 × 10^6^ cells in 100 μl of sterilized PBS) were injected into the right and left dorsal flanks of 4-week-old BALB/c male nude mice (Animal Center of the Chinese Academy of Science, Shanghai, China), respectively. Next, all mice were raised in a pathogen-free condition. The lengths and widths of the tumors were measured every week, and the tumor volume was calculated as follows: tumor volume = (length × width^2^)/2. At 5 weeks after injection, all mice were euthanized, and their tumors were dissected. The tumors were subjected to IHC staining to analyze the expression levels of ANLN, EZH2 and LASP1 with primary antibodies against ANLN (1:200; Abcam, Cambridge, United Kingdom), EZH2 (1:200; Abcam, Cambridge, United Kingdom) and LASP1 (1:200; Proteintech, China).

### Microarray analysis

BxPC-3 cells were transfected with NC or ANLN RNAi and collected after 3 days, and three biological replicates were utilized. Total RNA was extracted using TRIzol reagent (Invitrogen, Grand Island, NY, USA). The RNA quality was determined by a spectrophotometer at 260 and 280 nm. The RNA integrity was evaluated by electrophoresis (1% formaldehyde denaturing gel). The RNA was subsequently synthesized into cDNA, and converted into cRNA. Labeled cRNA was hybridized to the Affymetrix Gene Chip Human Gene 1.0 ST Array (Affymetrix, Santa Clara, CA, USA). Expression Console and Transcriptome Analysis Console v3.0 (Affymetrix, USA) were used to analyze differentially expressed genes. Gene ontology (GO) annotation analysis was performed using DAVID Bioinformatics Resources 6.8 (https://david.ncifcrf.gov/).

### Quantitative reverse transcription polymerase chain reaction (qRT-PCR)

Total RNA was isolated from cells using TRIzol reagent (Invitrogen, USA), according to manufacturer’s instructions. A spectrophotometer was used to determine the RNA quality and quantity. RNA was then reverse-transcribed into cDNA using M-MLV Reverse Transcriptase (TaKaRa, Dalian, China) and BulgeLoop™ specific RT-primers and random primers for mRNA. The gene expression levels were analyzed using a SYBR Premix Ex Taq kit (TaKaRa, China). The relative expression of gene was calculated using the 2^-ΔΔCt^ method. β-actin or U6 was used as an internal control. This experiment was performed with three biological replicates. All primer sequences are listed in Additional file 1: Table S1.

### Luciferase reporter assay

The partial wild-type sequence of the LASP1 3′-untranslated region (UTR) (2262 bp) containing the three putative miR-218-5p binding sites (Site1:686–692, Site2: 1587–1593 and Site3 2080–2087) or the sequences having mutations of the miR-218-5p putative binding sites in LASP1 3’UTR were cloned into the downstream of the luciferase gene in the psiCHECK-2 vector (Promega, Madison, WI, USA).

BxPC-3 and SW1990 cells were cotransfected with the LASP1 3’UTR luciferase expression plasmid and the miR-218-5p mimic or con using Lipofectamine 2000 (Invitrogen). After 48 h of transfection, a dual-luciferase reporter assay system (Promega, Madison, WI, USA) was used to determine the luciferase activity. The firefly luciferase activity was normalized to *Renilla* activity.

### Statistical analysis

Data from Western blot, cell proliferation, colony formation, cell migration, cell invasion, qRT-PCR, Luciferase reporter and in vivo tumor growth assays were analyzed using SPSS17.0 statistical software (IBM Corporation, Armonk, NY, USA) and presented as an average of biological replicates (mean ± S.D.). Student’s t-test or one-way ANOVA was used to evaluate the differences. Associations between ANLN expression and the clinicopathologic parameters were determined by the non-parametric Pearson Chi-Square test. The survival rates for each variable were analyzed using the Kaplan-Meier method. Moreover, log-rank statistics were used to estimate the equivalences of the survival curves. The parameters with statistical significance in the univariate survival analysis were subjected to further evaluation via multivariate survival analysis. *P* values < 0.05 were considered to be statistically significant.

## Results

### ANLN expression was upregulated in pancreatic cancer tissues and cell lines

**Fig. 1:**
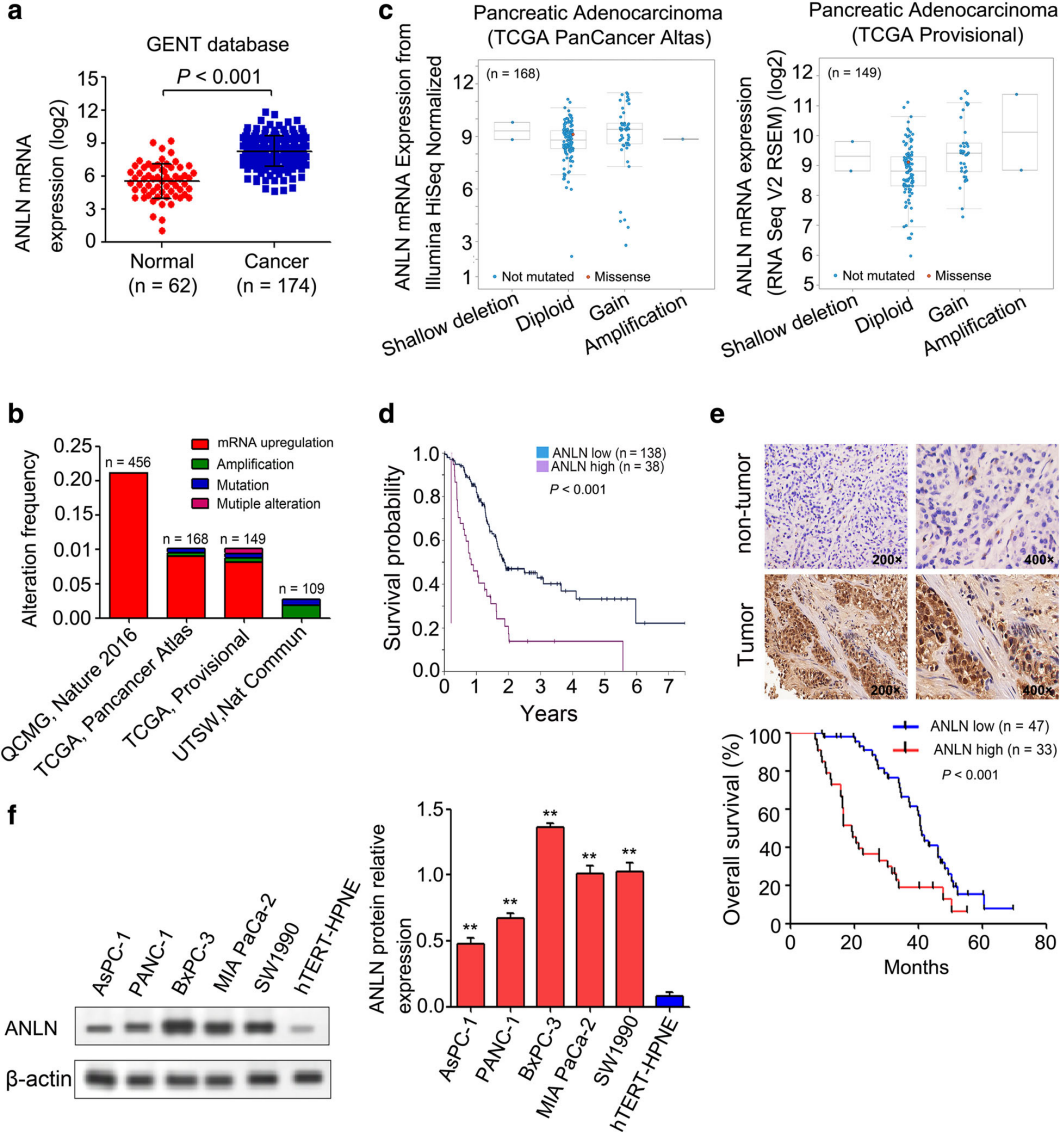
ANLN was upregulated in pancreatic cancer. **a**, ANLN expression was significantly upregulated in 174 pancreatic cancer tissues compared with that in 62 normal tissues (*P* < 0.001, http://medical-genome.kribb.re.kr/GENT/). **b**, The genetic alteration frequency of ANLN was evaluated in four independent cohorts (QCMG, Nature 2016; TCGA, PanCancer Atlas; TCGA, Provisional; UTSW, Nat Commun) from cBioportal for Cancer Genomic (www.cbioportal.org/). **c**, Genetic abnormalities of the copy number of the ANLN gene were plotted in two pancreatic cancer datasets (Pancreatic Adenocarcinoma-TCGA, PanCancer Atlas and Pancreatic Adenocarcinoma-TCGA, Provisional) from cBioportal for Cancer Genomic (www.cbioportal.org/). **d**, The association between ANLN expression in pancreatic cancers and survival time was analyzed by Kaplan–Meier survival analysis in the Human Protein Atlas (www.proteinatlas.org/). **e**, Representative photomicrographs from ANLN IHC staining of eighty pancreatic cancer tissues and ten cancer-adjacent normal tissues are shown in the upper panel (× 200 and × 400 magnification). Kaplan-Meier survival curve of the overall survival according to high and low ANLN expression in pancreatic cancer tissues is shown in the lower panel (*P* < 0.001). **f**, The ANLN protein expression levels were increased in five pancreatic cancer cell lines (AsPC-1, PANC-1, BxPC-3, MIA PaCa-2 and SW1990) compared with those in the hTERT-HPNE cell line. **, *P* < 0.01 compared with the hTERT-HPNE cell line

According to the GENT database, ANLN expression was significantly upregulated in 174 pancreatic cancer tissues compared with that in 62 normal tissues (*P* < 0.001, Fig. 1a) [39]. In addition, results from cBioportal for Cancer Genomic (www.cbioportal.org/) showed that the mRNA upregulation of the ANLN gene accounted for most of the alterations (QCMG, Nature 2016; TCGA, Pancancer Atlas; TCGA, Provisional; UTSW, Nat Commun) (Fig. 1b) [40, 41]. Moreover, fewer ANLN shallow deletion and high-level ANLN gene amplification (amplification) and more ANLN diploid and low-level ANLN gene amplification (gain) were observed in two pancreatic cancer datasets (Pancreatic Adenocarcinoma-TCGA, PanCancer Atlas and Pancreatic Adenocarcinoma-TCGA, Provisional) (Fig. 1c). The ANLN mRNA expression in the pancreatic cancer samples with low-level ANLN gene amplification (gain) was markedly increased compared with that in the pancreatic cancer samples with ANLN diploids (Fig. 1c). In addition, Kaplan-Meier plots summarizing the result from the Human Protein Atlas (www.proteinatlas.org/) showed that the upregulation of ANLN was significantly correlated with worse overall survival (*P* = 0.000002, Fig. 1d). Consistent with these publicly available data, our results also showed significantly upregulated ANLN expression in pancreatic cancer tissues compared with that in their matched adjacent normal pancreatic tissues (Fig. 1e). We subsequently analyzed the association between the ANLN expression and clinicopathological features in 80 pancreatic cancer samples. We found that ANLN upregulation was significantly correlated with the tumor size (*P* = 0.002), tumor differentiation (*P* = 0.027), TNM stage (*P* < 0.001), lymph node metastasis (*P* = 0.004) and distant metastasis (*P* = 0.005) (Table 1). In addition, univariate analysis indicated that the TNM stage, lymph node metastasis and upregulated ANLN expression were associated with overall survival (*P* = 0.002, *P* = 0.016 and *P* = 0.034, respectively) (Table 2). Multivariate analysis showed that the TNM stage, lymph node metastasis and upregulated ANLN expression were independent prognostic factors for overall survival (*P* = 0.001, *P* = 0.021 and *P* = 0.002, respectively) (Table 2). Kaplan-Meier analysis showed that upregulated ANLN expression was significantly associated with shorter survival for patients with pancreatic cancer (*P* = 0.001, Fig. 1e). Moreover, we analyzed the ANLN protein expression in one normal human pancreatic duct epithelial cell line (hTERT-HPNE) and five pancreatic cancer cell lines (AsPC-1, PANC-1, BxPC-3, MIA PaCa-2 and SW1990). Our results showed that ANLN protein expression was significantly increased in the five pancreatic cancer cell lines compared with that in the hTERT-HPNE cell line (Fig. 1f).

**Table 1:** Correlations between ANLN expression and clinicopathological characteristics of patients with pancreatic cancer

Variable	All cases	ANLN		*P* value
		Low expression	High expression	
Age				0.429
≤ 60	37	20	17	
> 60	43	27	16	
Gender				
Male	46	26	20	0.638
Female	34	21	13	
Tumor size (cm)				
< 2	41	31	10	0.002**
≥ 2	39	16	23	
Tumor differentiation				
Well	36	26	10	0.027*
Poor	44	21	23	
TNM stage				
I + II	25	22	3	0.000**
III + IV	55	25	30	
Lymph node metastasis				
Negative	49	35	14	0.004**
Positive	31	12	19	
Distant metastasis				
M0	53	37	16	0.005**
M1	27	10	17	

*Statistically signi*fi*cant (*P* < 0.05), **Statistically signi*fi*cant (*P* < 0.01)

**Table 2:** Cox proportional hazard models for prognostic factors

	Univariate analysis		Multivariate analysis	
	HR(95% CI)	*P* value	HR(95% CI)	*P* value
Age (> 60 vs. ≤60)	1.371 (0.749–2.510)	0.306		
Gender (famale vs. male)	0.874 (0.491–1.557)	0.648		
Tumor size (≥2 vs. < 2)	1.502 (0.836–2.699)	0.173		
Tumor differentiation (Poor vs. Well)	1.287 (0.738–2.244)	0.375		
TNM stage (III + IV vs. I + II)	3.013 (1.495–6.073)	0.002**	3.043 (1.542–6.004)	0.001**
Lymph node metastasis (Positive vs. Negative)	2.109 (1.151–3.864)	0.016*	1.873 (1.100–3.188)	0.021*
Distant metastasis (M1 vs. M0)	1.244 (0.679–2.280)	0.479		
ANLN expression (high vs. low)	1.923 (1.051–3.521)	0.034*	2.352 (1.369–4.039)	0.002**

*Statistically signi*fi*cant (*P* < 0.05), **Statistically signi*fi*cant (*P* < 0.01)

### ANLN downregulation inhibited pancreatic cancer tumorigenesis in vitro and in vivo

**Fig. 2:**
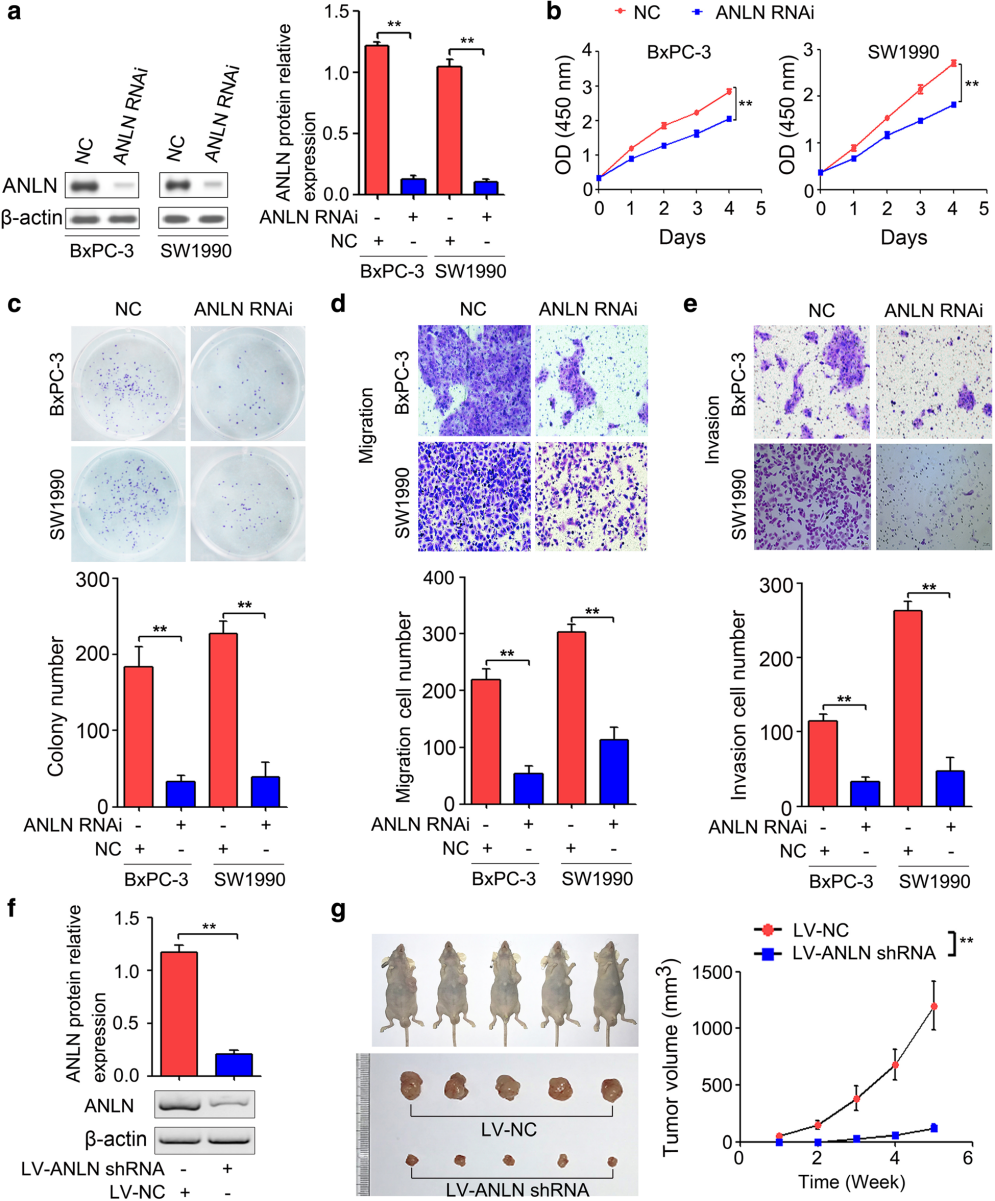
ANLN functioned as an oncogene in pancreatic cancer. **a**, The ANLN protein expression levels in BxPC-3 and SW1990 cells transfected with ANLN siRNA (ANLN RNAi) or the scramble control (NC) were analyzed by Western blot. **b**, Proliferation of BxPC-3 and SW1990 cells transfected with ANLN siRNA (ANLN RNAi) or the scramble control (NC) was determined by the CCK-8 assay. **c**, ANLN knockdown significantly inhibited the colony forming ability of BxPC-3 and SW1990 cells. **d** and **e**, The migration and invasion properties of the same cells described in **b** were determined by the transwell migration and invasion assay. **f**, Knockdown effect of ANLN silencing lentiviral vectors was confirmed by Western blot. **g**, ANLN knockdown in BxPC-3 cells repressed the tumor growth. *n* = 5 per group. **P < 0.01

To determine the function of ANLN in pancreatic cancer, we conducted siRNA-mediated gene silencing. As shown in Fig. 2a, ANLN expression was significantly reduced in the BxPC-3 and SW1990 cell lines after transfection with ANLN siRNA (ANLN RNAi) compared with that in the negative control group (NC) (*P* < 0.01). After confirming the suppressive effect of ANLN siRNA by Western blot, we performed CCK-8 assays and showed that ANLN downregulation significantly inhibited BxPC-3 and SW1990 cell proliferation when compared with that in the NC group (Fig. 2b). In the colony formation assay, the colony numbers were significantly decreased in cells transfected with ANLN siRNA compared with those transfected with the control (Fig. 2c). The migration and invasion assay indicated that knockdown of ANLN significantly suppressed cell migration and invasion (Fig. 2d and e). Moreover, we established the stable ANLN silencing BxPC-3 cell line using lentiviral vectors with puromycin. The knockdown effect of ANLN-silencing lentiviral vectors was confirmed by Western blot. The ANLN-silencing lentiviral vectors (LV-ANLN shRNA) clearly inhibited the ANLN protein expression in BxPC-3 cells compared with that resulting from the control lentiviral vectors (LV-NC) (Fig. 2f). Moreover, the xenograft tumor volumes of the LV-ANLN shRNA group were obviously reduced when compared with those of the LV-NC group (*P* < 0.01, Fig. 2g).

### Downregulation of ANLN in BxPC-3 cells exerted strong effects on gene expression

**Fig. 3:**
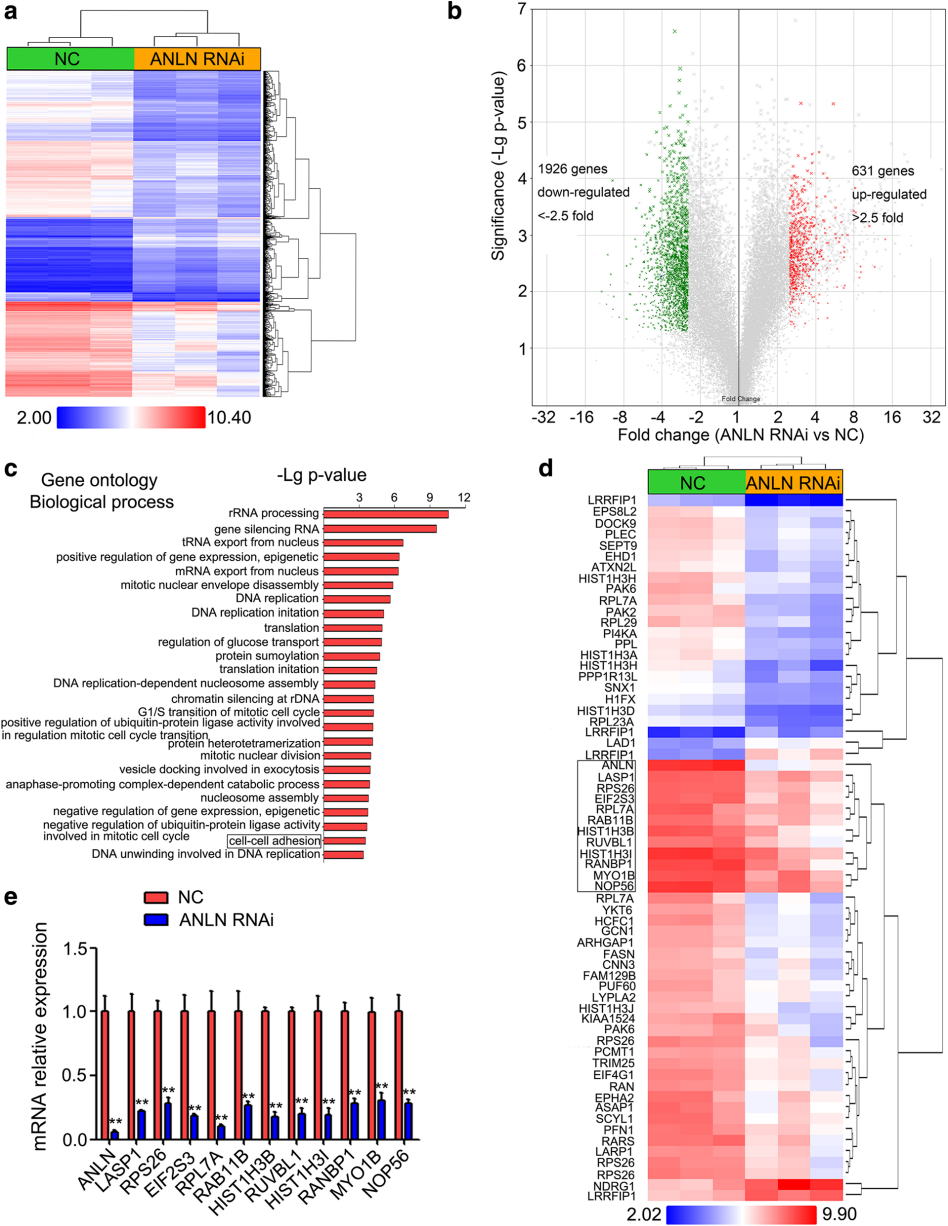
Global changes in gene expression in BxPC-3 cells transfected with ANLN siRNA. **a**, The heat map of the differentially expressed genes with a fold change of greater than 2.5 or less than − 2.5 in ANLN RNAi relative to NC. **b**, A volcano plot showing the differentially expressed genes with a fold change of greater than 2.5 or less than − 2.5 in ANLN RNAi relative to NC. **c**, The top twenty-five regulated Gene ontology (GO) biological process terms. **d**, The heat map of 54 cell-cell adhesion-related genes. **e**, The selected candidate genes from microarray experiments were confirmed by qRT-PCR after ANLN RNAi transfection in BxPC-3 cells. **P < 0.01 compared with the NC group

To explore the potential mechanisms of ANLN in regulating pancreatic cancer progression, gene microarray analysis was performed using RNA isolated from BxPC-3 cells transfected with NC or ANLN RNAi. A total of 1926 genes were downregulated with fold changes < − 2.5 (*P* < 0.05), while 631 genes were upregulated with fold changes > 2.5 (*P* < 0.05) (Fig. 3a and b). The downregulated and upregulated genes were subsequently submitted to gene ontology (GO) annotation analysis using the DAVID Bioinformatics Resources 6.8 (https://david.ncifcrf.gov/). The Go terms representing biological processes indicated that these differentially expressed genes were highly enriched in cell-cell adhesion and cell cycle-related biological processes (Additional file 2: Table S2, Fig. 3c). The GO terms representing cellular compartment and molecular functions were shown in Additional file 3: Table S3 and Additional file 4: Table S4. Interestingly, cell-cell adhesion-related genes are largely involved in regulating cell growth, proliferation and motility [42, 43]. Based on GO analysis, 54 cell-cell adhesion-related genes were shown in Fig. 3d. According to the clustering results shown in Fig. 3d, eleven candidate genes were selected. To validate the microarray results, qRT-PCR was used to determine the expression of the eleven genes. Similar to the microarray results, the expression of all eleven genes in BxPC-3 cells transfected with ANLN RNAi was significantly decreased compared with that in the NC group (Fig. 3e).

### Upregulation of LASP1 contributed to the aggressive properties regulated by ANLN

**Fig. 4:**
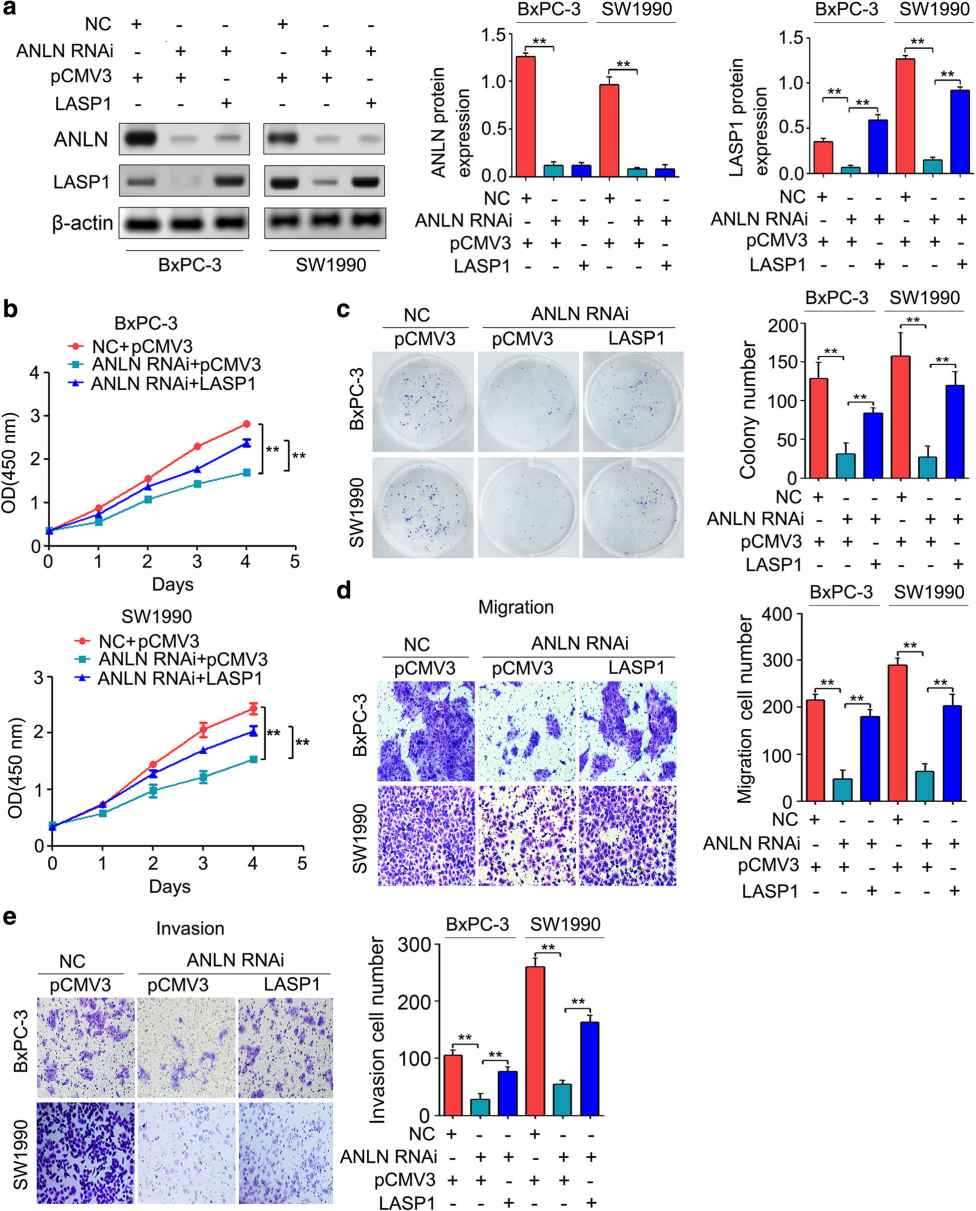
LASP1 re-expression partially reversed the effects of ANLN knockdown on proliferation, colony formation, migration and invasion in pancreatic cancer cells. **a**, LASP1 expression vectors rescued LASP1 expression in BxPC-3 and SW1990 cells transfection with ANLN RNAi. **b**, CCK-8 analysis indicated that LASP1 re-expression reversed the suppressive effects of ANLN knockdown on pancreatic cancer cell growth. **c**, The restoration of LASP1 expression reversed the suppressive effects of ANLN knockdown in colony formation. **d** and **e**, The restoration of LASP1 expression reversed the suppressive effects of ANLN knockdown in migration and invasion. **P < 0.01

Among the eleven candidate genes selected according to the clustering results, LASP1, RAB11B, RUVBL1 and MYO1B are upregulated in human malignant tumors and contribute to cancer progression [20, 44–46]. Based on the GENT database, we showed that both LASP1 and RUVBL1 gene expression were significantly upregulated in pancreatic cancer tissues compared with that in normal pancreatic tissues, while RAB11B was not changed obviously, and MYO1B was significantly downregulated (Additional file 5: Figure S1A). In addition, both LASP1 and RUVBL1 expression were positively correlated with ANLN expression (Additional file 5: Figure S1B). Further investigation showed that LASP1 restoration partially reversed the effects of ANLN knockdown on pancreatic cancer cell proliferation (Additional file 5: Figure S1C). However, RUVBL1 restoration did not reverse the effect of ANLN downregulation on pancreatic cancer cell proliferation (Additional file 5: Figure S1C). Thus, ANLN may promote pancreatic cancer cell progression by regulating LASP1. To confirm this hypothesis, we rescued LASP1 expression using LASP1 overexpression plasmid vectors in the cells transfected with ANLN RNAi. As shown in Fig. 4a, the LASP1 protein expression in ANLN RNAi-transfected cells was restored by the LASP1 plasmid. In addition, the CCK-8 and colony formation assays indicated that the suppressive effects of ANLN knockdown on pancreatic cancer cell growth were restored by LASP1 re-expression (Fig. 4b and c). Moreover, LASP1 re-expression partially reversed the effect of ANLN knockdown on pancreatic cancer cell migration and invasion (Fig. 4d and e).

### LASP1 is a crucial target of miR-218-5p in pancreatic cancer

**Fig. 5:**
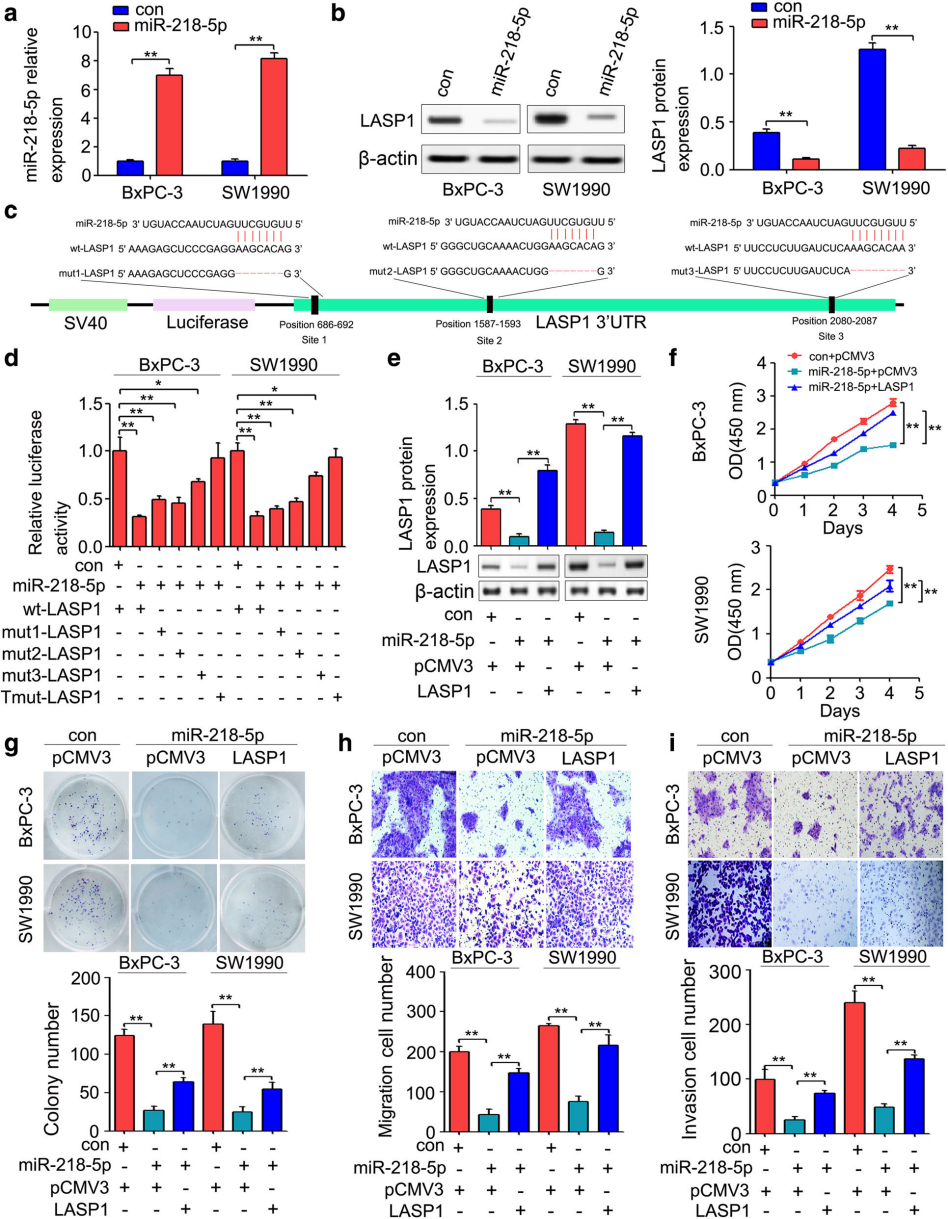
MiR-218-5p inhibited pancreatic cancer cell progression by directly regulating LASP1. **a**, The expression levels of miR-218-5p were detected by qRT-PCR in BxPC-3 and SW1990 cells transfected with the miR-218-5p mimic (miR-218-5p) or mimic control (con). U6 was used as a loading control. **b**, The expression levels of LASP1 protein in BxPC-3 and SW1990 cells transfected with the miR-218-5p mimic (miR-218-5p) or mimic control (con) were analyzed by Western blot. **c**, Representation of the seed pairing between miR-218-5p and LASP1 3’UTR. Three mutations (mut1-LASP1, mut2-LASP1 and mut3-LASP1) were generated for luciferase assay in the sequence complementary to the miR-218-5p target binding region. **d**, BxPC-3 and SW1990 cells were co-transfected with either miR-218-5p mimic (miR-218-5p) or mimic control (con) and the wild-type or mutant LASP1 3’UTR luciferase reporter vector for 48 h.The relative luciferase activity was determined by the dual-luciferase assay. *Renilla* luciferase activity was used as a loading control. **e**, Efficiency of LASP1 re-expression was determined by Western blot. **f**, CCK-8 analysis revealed that LASP1 re-expression partly reversed the growth repression of miR-218-5p on pancreatic cancer cells. **g**, The restoration of LASP1 expression reversed the suppressive effects of miR-218-5p in colony formation. **h** and **I**, Ectopic expression of LASP1 reversed the suppressive effects of miR-218-5p in migration and invasion. *P < 0.05, **P < 0.01

Previous reports have shown that microRNAs (miRNAs) play an important role in cancer initiation and tumor progression by directly regulating target genes expression [47]. Here, we found that a total of 49 miRNA precursors showed significant changes in gene expression profiles; 46 miRNA precursors were upregulated, and 3 miRNA precursors were downregulated (Additional file 6: Figure S2A). By combining the gene expression profiles and Targetscan (http://www.targetscan.org/), three miRNAs (miR-145-5p, miR-218-5p and miR-9-5p) were found both upregulated in ANLN downregulated BxPC-3 cells and contained binding sites of the 3’UTR of LASP1 (Additional file 6: Figure S2B). To determine the effect of miR-145-5p, miR-218-5p and miR-9-5p on LASP1 expression, miR-145-5p, miR-218-5p and miR-9-5p mimics were transfected into BxPC-3 cells, respectively. We found that the expression of miR-145-5p, miR-218-5p and miR-9-5p in transfected cells was significantly upregulated (Additional file 6: Figure S2C). Additional qRT-PCR showed that only miR-218-5p upregulation significantly repressed LASP1 mRNA expression in BxPC-3 cells (Additional file 6: Figure S2D). To further confirm the effect of miR-218-5p on LASP1 expression, the mimic control (con), miR-218-5p mimics (miR-218-5p) were transfected into BxPC-3 and SW1990 cells, respectively. As shown in Fig. 5a, the expression of miR-218-5p in transfected cells was significantly upregulated. Moreover, miR-218-5p upregulation significantly suppressed LASP1 protein expression in BxPC-3 and SW1990 cells (Fig. 5b). To determine whether miR-218-5p directly targets the 3’UTR of LASP1, the luciferase reporter vectors containing the 3’UTR of LASP1 (wt-LASP1) or its mutant version were constructed (Fig. 5c). We found that the luciferase activity of miR-218-5p-transfected cells was significantly inhibited compared with that of the con-transfected cells. In addition, site-directed mutagenesis of the miRNA binding sequences in the 3’UTR of LASP1 showed a slightly different trend. The conserved miRNA binding site (Site3) appeared to be slightly more effective in miR-218b-5p-induced luciferase activity repression because mutation of this site significantly reversed a decrease in the luciferase signal (Fig. 5d). However, the miR-218-5p-mediated repression of luciferase activity was abolished by mutating the three binding sequences (triple mutation) of the LASP1 3’UTR (Fig. 5d). To further determine the role of LASP1 in the suppressive effects of miR-218-5p on pancreatic cancer progression, we rescued the expression of LASP1 by LASP1 overexpression plasmid vectors in the cells transfected with miR-218-5p. As shown in Fig. 5e, LASP1 protein expression in miR-218-5p-transfected cells was restored by LASP1 plasmid. In addition, CCK-8 analysis and colony formation assay revealed that the suppressive effects of miR-218-5p upregulation in pancreatic cancer cell growth were partially restored by LASP1 re-expression (Fig. 5f and g). Moreover, LASP1 re-expression partially reversed the inhibition of cell migration and invasion caused by miR-218-5p upregulation (Fig. 5h and i).

### miR-218-5p was responsible for the ANLN-induced LASP1 expression and pancreatic cancer cell growth, migration and invasion

**Fig. 6:**
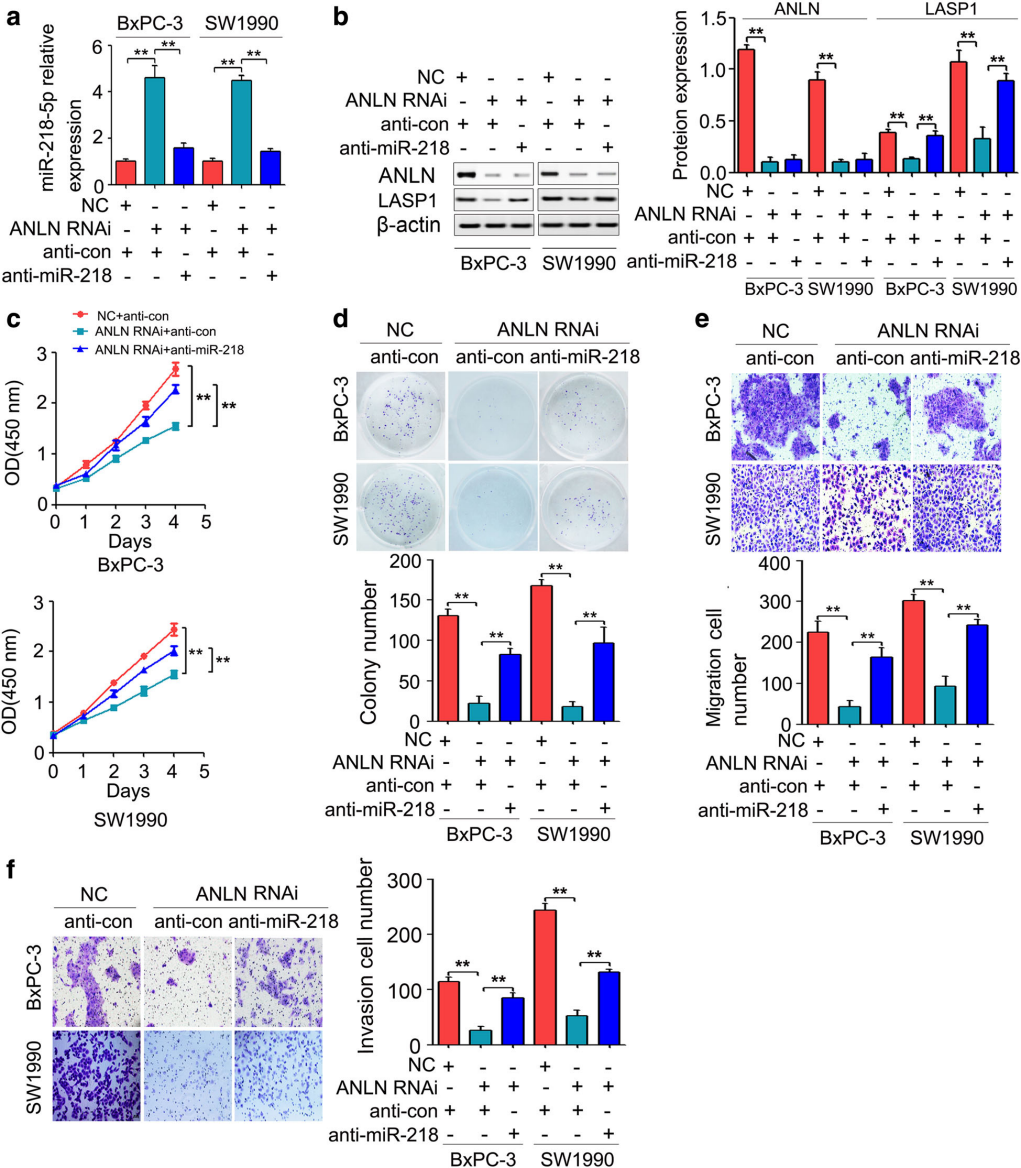
MiR-218-5p was involved in the ANLN-induced LASP1 expression and pancreatic cancer progression. **a**, MiR-218-5p inhibitor (anti-miR-218) obviously reversed the ANLN knockdown-induced miR-218-5p expression. **b**, The LASP1 protein levels were partly restored in the cells cotransfected with ANLN RNAi and miR-218-5p inhibitor (anti-miR-218) compared with the protein levels in the cells cotransfected with ANLN RNAi and inhibitor control (anti-con). **c**, CCK-8 analysis revealed that miR-218-5p inhibitor (anti-miR-218) rescued the inhibition of cell proliferation in BxPC-3 and SW1990 cells transfected with ANLN RNAi. **d**, Partial restoration of the suppressed cell growth was observed by colony formation after miR-218-5p inhibition. **e** and **f**, miR-218-5p inhibition in BxPC-3 and SW1990 cells partly reversed the suppressive effects of ANLN knockdown in migration and invasion. **P < 0.01

In this study, we showed that miR-218-5p upregulation inhibited pancreatic cancer cell growth, migration and invasion by directly regulating LASP1 expression. Moreover, ANLN knockdown significantly induced the expression of miR-218-5p. Thus, ANLN may regulate LASP1 expression and pancreatic cancer progression by miR-218-5p. To determine whether miR-218-5p was involved in ANLN-induced LASP1 expression and pancreatic cancer cell growth, migration and invasion, miR-218-5p inhibitor (anti-miR-218) was used to reverse the expression of miR-218-5p upregulation caused by ANLN knockdown. As shown in Fig. 6a, anti-miR-218 obviously reversed the ANLN knockdown-induced miR-218-5p expression. In addition, the LASP1 protein levels were restored in the cells cotransfected with ANLN RNAi and anti-miR-218 compared with the protein levels in the cells cotransfected with ANLN RNAi and inhibitor control (anti-con) (Fig. 6b). In functional assays, miR-218-5p knockdown in pancreatic cancer cells transfected with ANLN RNAi rescued the inhibition of cell proliferation, colony formation, cell migration and cell invasion caused by ANLN knockdown (Fig. 6c-f). Collectively, these results demonstrated that ANLN promotes pancreatic cancer cell growth, migration and invasion by regulating miR-218-5p/LASP1 signaling axis.

### EZH2 was involved in ANLN-induced pancreatic cancer cell growth, migration and invasion by mediating miR-218-5p/LASP1 signaling axis

**Fig. 7:**
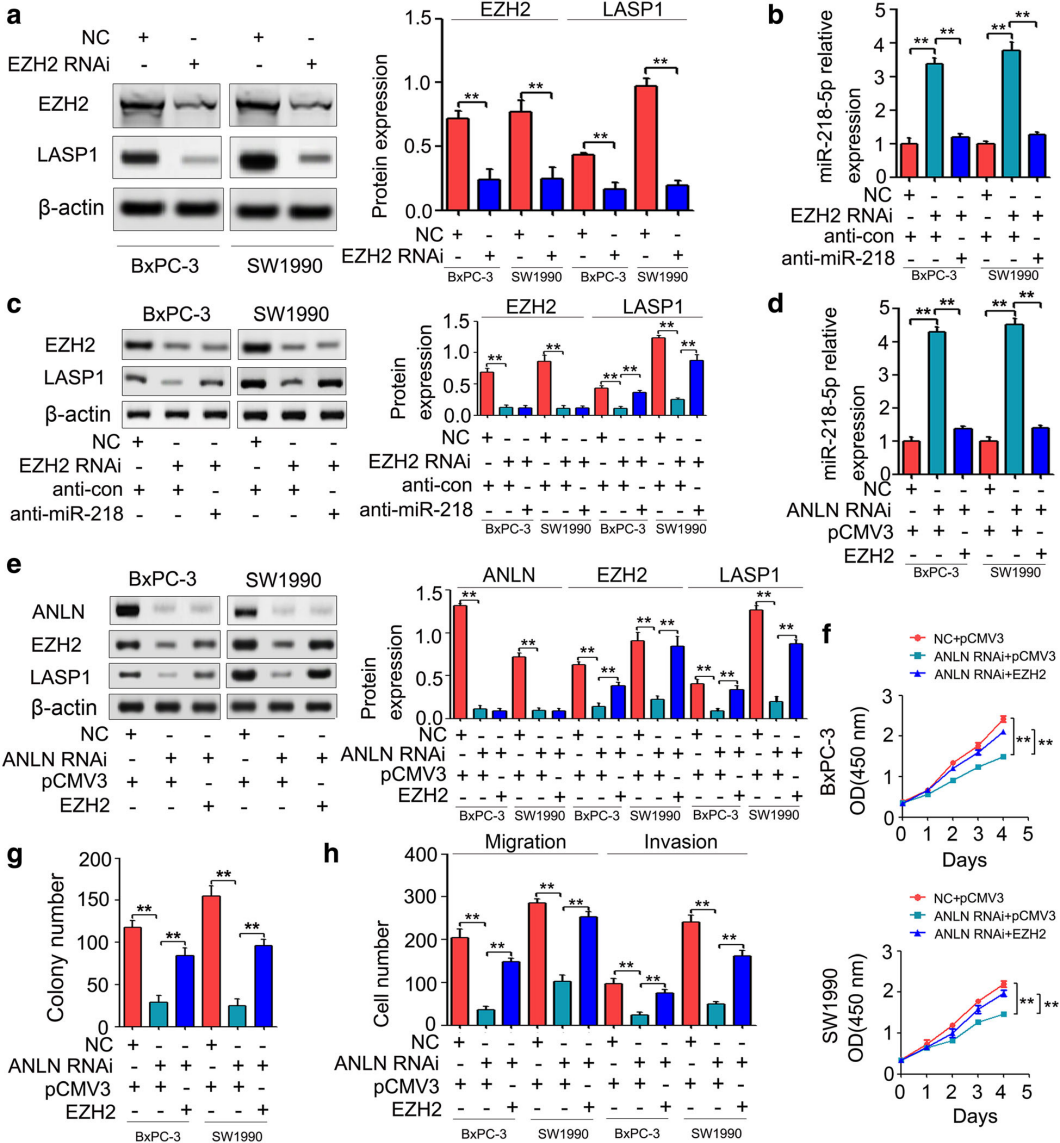
EZH2 was responsible for ANLN-induced pancreatic cancer progression by mediating miR-218-5p/LASP1 signaling axis. **a**, EZH2 downregulation significantly inhibited the expression of LASP1 protein in BxPC-3 and SW1990 cells. **b**, MiR-218-5p inhibitor (anti-miR-218) obviously reversed the EZH2 knockdown-induced miR-218-5p expression. **c**, The LASP1 protein levels were partly restored in the cells cotransfected with EZH2 RNAi and anti-miR-218 compared with the protein levels in the cells cotransfected with EZH2 RNAi and inhibitor control (anti-con). **b**, EZH2 re-expression obviously reversed the ANLN knockdown-induced miR-218-5p expression. **e**, EZH2 re-expression obviously reversed the expression levels of LASP1 protein impaired by ANLN knockdown. **f**, CCK-8 analysis revealed that ectopic expression of EZH2 reversed the inhibition of cell proliferation in BxPC-3 and SW1990 cells transfected with ANLN RNAi. **g**, Partial restoration of the suppressed cell growth was observed by colony formation after LASP1 re-expression. **h**, Ectopic expression of EZH2 in BxPC-3 and SW1990 cells partly reversed the suppressive effects of ANLN knockdown in migration and invasion. **P < 0.01

In pancreatic cancer, EZH2 is upregulated and induces pancreatic cancer cell proliferation, cell migration and cell invasion in vitro and tumor growth and metastasis in vivo by inhibiting the expression of miR-218-5p [37]. In this study, the result of gene expression profiles showed that ANLN knockdown significantly inhibited the expression of EZH2 (Additional file 7: Figure S3A). To further confirm the effect of ANLN knockdown on EZH2, qRT-PCR and Western blot analysis was performed. Our results showed that ANLN knockdown significantly repressed the expression of EZH2 mRNA and protein (Additional file 7: Figure S3B and C). Therefore, ANLN may promote pancreatic cancer cell progression and miR-218-5p/LASP1 signaling axis by mediating EZH2. To determine whether EZH2 regulates LASP1, we transfected the BxPC-3 and SW1990 cells with the negative control siRNA (NC) or EZH2 siRNA (EZH2 RNAi). As shown in Fig. 7a, EZH2 downregulation significantly inhibited the expression of LASP1 protein. In addition, to further confirm whether EZH2 downregulation could inhibit LASP1 expression by inducing miR-218-5p expression, miR-218-5p inhibitor (anti-miR-218) was used to reverse the EZH2 knockdown-induced miR-218-5p expression. As shown in Fig. 7b, anti-miR-218 obviously reversed the EZH2 knockdown-induced miR-218-5p expression. Moreover, the LASP1 protein levels were restored in the cells cotransfected with EZH2 RNAi and anti-miR-218 compared with the protein levels in the cells cotransfected with EZH2 RNAi and inhibitor control (anti-con) (Fig. 7c). To further determine whether EZH2 was involved in ANLN-induced LASP1 expression and the inhibition of miR-218-5p, EZH2 expression plasmid vectors were used to rescue the inhibition of EZH2 caused by ANLN knockdown. As shown in Fig. 7d, the ectopic expression of EZH2 reversed the upregulation of miR-218-5p expression caused by ANLN knockdown. In addition, EZH2 re-expression reversed the inhibition of LASP1 caused by ANLN knockdown (Fig. 7e). In functional assays, EZH2 restoration in pancreatic cancer cells transfected with ANLN RNAi rescued the inhibition of cell proliferation, colony formation, cell migration and cell invasion caused by ANLN knockdown (Fig. 7f-h).

### Downregulation of ANLN contributed to the inhibition of EZH2 and LASP1 in vivo

**Fig. 8:**
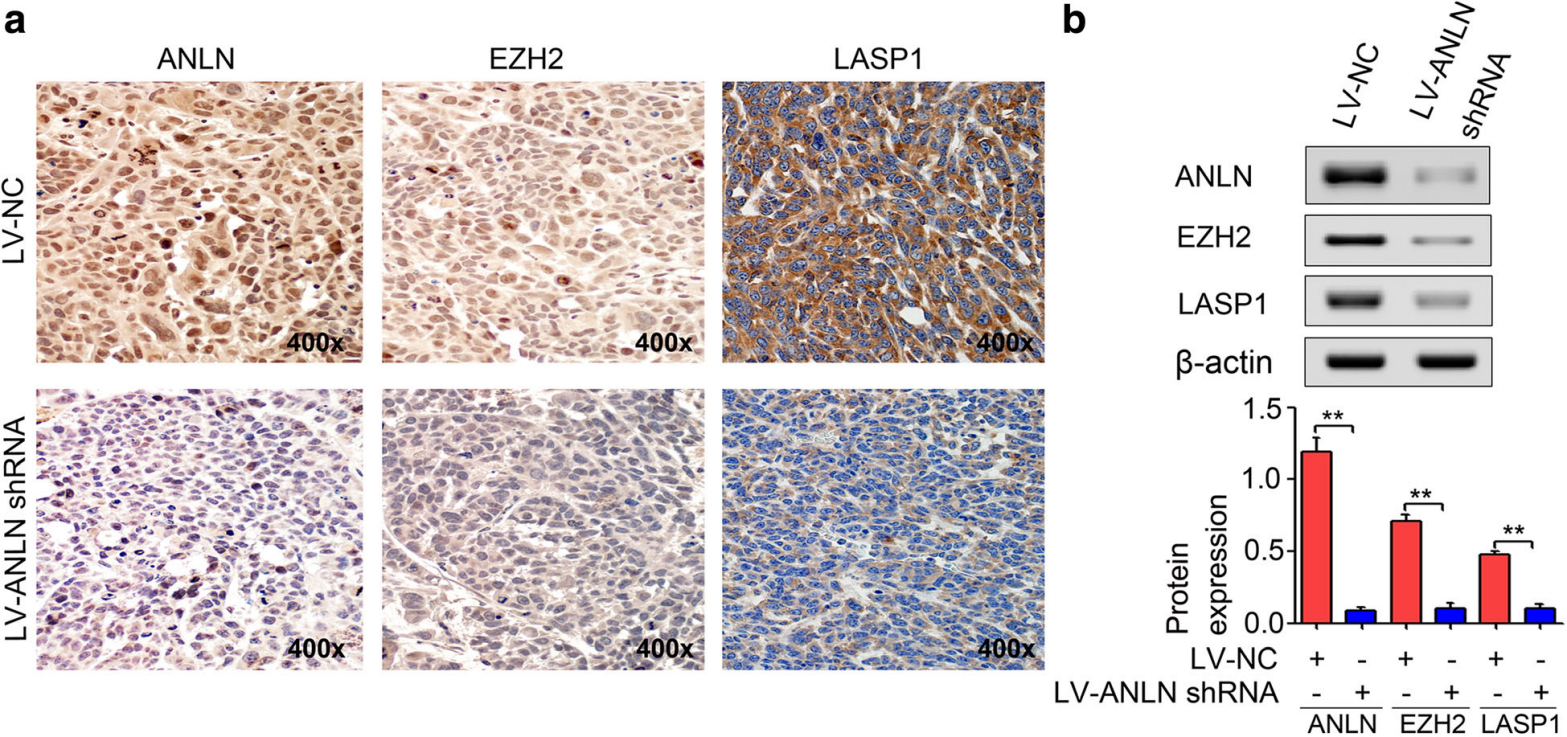
ANLN knockdown repressed the levels of EZH2 and LASP1 expression in vivo. **a**, The levels of ANLN, EZH2 and LASP1 expression in tissues from xenograft tumor models established with the stable ANLN-silenced BxPC-3 cells or the stable scramble control BxPC-3 cells were determined by IHC. **b**, Western blot analysis in tumors as in (**a**). **P < 0.01

**Fig. 9:**
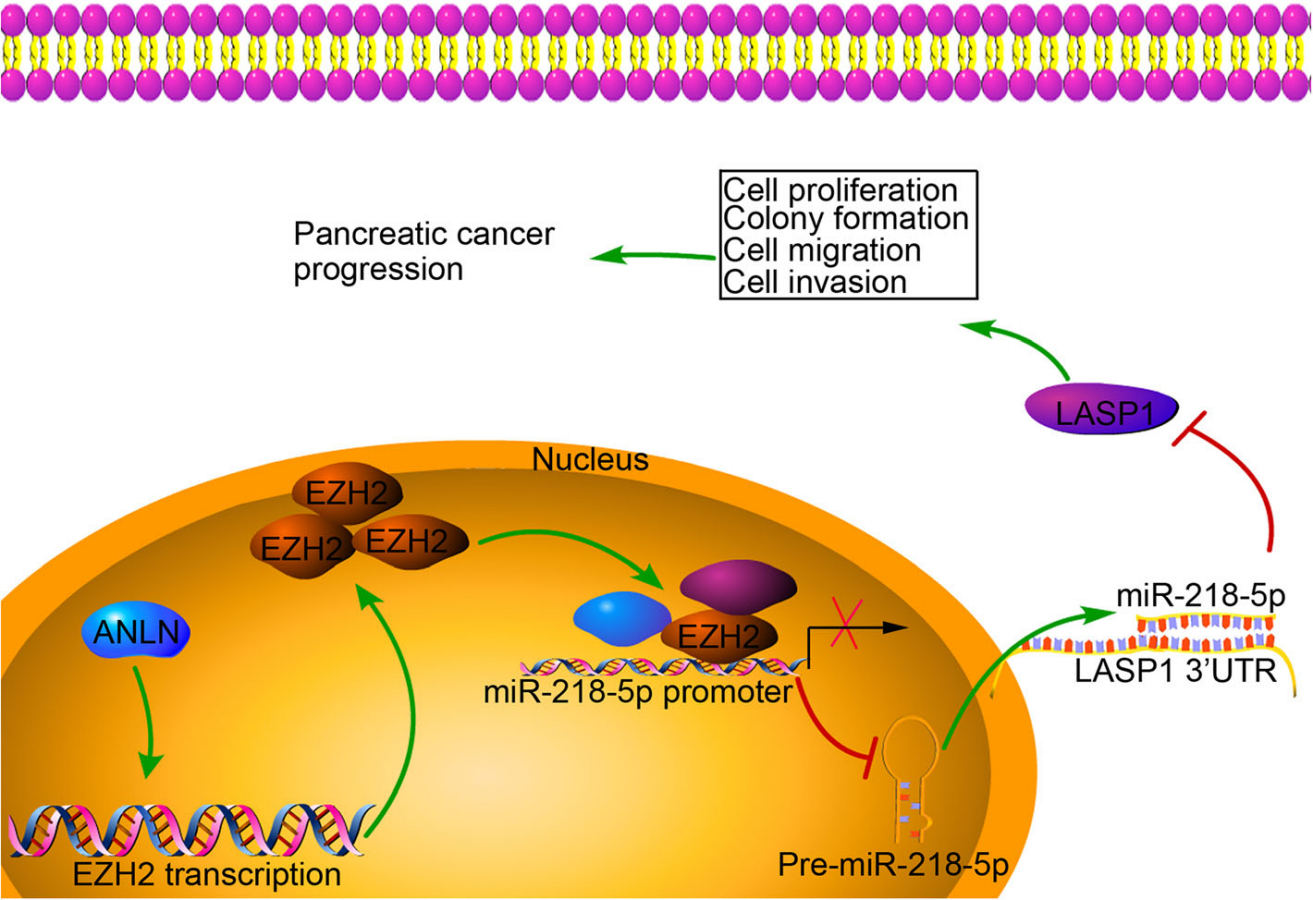
Schematic diagram for the effect of ANLN on pancreatic cancer progression. ANLN upregulation promotes cell proliferation, colony formation, migration and invasion by inducing EZH2. ANLN-induced EZH2 expression leads to the downregulation of miR-218-5p, which inhibits LASP1 expression by directly targeting the 3’UTR of LASP1

To confirm the effect of ANLN knockdown on the expression of EZH2 and LASP1, we detected the levels of ANLN, EZH2 and LASP1 expression in tissues from in vivo xenograft tumor models established with the stable ANLN-silenced BxPC-3 cells or the stable scramble control BxPC-3 cells by IHC and Western blot. IHC staining showed that the levels of EZH2 and LASP1 expression were repressed after ANLN knockdown (Fig. 8a). Consistent with these findings, Western blot results also showed that the levels of EZH2 and LASP1 expression were significantly inhibited in the LV-ANLN-shRNA group when compared to the LV-NC group (Fig. 8b). Taken together, ANLN may promote pancreatic cancer cell growth, migration and invasion by regulating EZH2/miR-218-5p/LASP1 signaling axis (Fig. 9).

## Discussion

The present study demonstrated that ANLN was upregulated in pancreatic cancer, and upregulated ANLN was associated with a poorer outcome in patients with pancreatic cancer. Our results also showed that ANLN downregulation inhibited cell proliferation, colony formation, migration and invasion. In addition, for the first time, we identified EZH2 as a downstream effector of ANLN that positively regulates EZH2 expression. EZH2 upregulation induced by ANLN promoted pancreatic cancer cell progression by miR-218-5p/LASP1 signaling axis. Thus, ANLN may be a useful prognostic indicator and an important therapeutic target in the treatment of pancreatic cancer.

Previous studies have shown that ANLN is upregulated in many cancers and may serve as a promising prognostic biomarker for cancer [13–18]. For example, ANLN was upregulated in bladder urothelial carcinoma, and elevated ANLN expression was correlated with poor progression-free and recurrence-free survival [16]. ANLN expression was increased in pancreatic cancer, and high ANLN expression was associated with a poor prognosis in the pancreatic ductal adenocarcinoma TCGA database [10, 18]. Consistent with previous reports, our results from the GENT database and IHC analysis showed that ANLN expression was significantly upregulated in pancreatic cancer tissues, and increased ANLN expression was significantly correlated with the tumor size, tumor differentiation, TNM stage, lymph node metastasis, distant metastasis and poorer outcome of patients with pancreatic cancer. Moreover, ANLN could serve as an independent predictor for overall survival of pancreatic cancer patients. In addition, we found that ANLN exerted tumorigenicity based on our functional studies. Consistent with our results, ANLN knockdown significantly repressed cell proliferation, migration and invasion in bladder urothelial carcinoma [16]. ANLN knockdown significantly inhibited the proliferation and migration and invasion of pancreatic cancer cells [13]. Thus, these results showed that therapeutic interventions based on ANLN regulatory strategies may be an effective way to prevent pancreatic cancer progression.

Gene microarray analysis in BxPC-3 cells transfected with ANLN siRNA showed that the genes with altered expression were highly enriched in the cell-cell adhesion and cell cycle-related biological processes (Additional file 2: Table S2, Fig. 3). It is well known that the molecules involved in cell-cell adhesion orchestrate large-scale tumor behaviors such as proliferation and invasion [42, 43]. For instance, the E3 ubiquitin ligase HUWE1 could induce cell migration and invasion by promoting cell-cell adhesion disassembly [48]. CD44 is largely involved in intracellular signaling for cell growth, proliferation and motility by mediating cellular adhesion [49]. Thus, cell-cell adhesion-related genes with altered expression in BxPC-3 cells after ANLN knockdown may play an important role in ANLN-induced pancreatic cancer cell progression. Based on GO and cluster analysis, eleven candidate genes with similar expressive trends and potential functions were selected and confirmed their expression by qRT-PCR (Fig. 3e). Among the eleven genes, four genes, including LASP1, RAB11B, RUVBL1 and MYO1B, are upregulated in human malignant tumors and promote cancer progression [20, 44–46]. Based on the GENT database, we showed that both LASP1 and RUVBL1 gene expression were significantly upregulated in pancreatic cancer tissues and were positively correlated with ANLN expression (Additional file 5: Figure S1A and B). Further investigation showed that only LASP1 restoration partially reversed the effects of ANLN knockdown on pancreatic cancer cell proliferation, colony formation, cell migration and cell invasion (Additional file 5: Figure S1C and Fig. 4). These results suggested that the upregulation of LASP1 expression induced by ANLN is partly responsible for pancreatic cancer progression.

Interestingly, our gene microarray analysis showed that 46 miRNA precursors were upregulated, and 3 miRNA precursors were downregulated (Additional file 6: Figure S2A). By combining the gene expression profiles and Targetscan (http://www.targetscan.org/), three miRNAs, including miR-145-5p, miR-218-5p and miR-9-5p, were selected and were found upregulated in ANLN downregulated BxPC-3 cells and contained binding sites of the 3’UTR of LASP1 (Additional file 6: Figure S2B), and mostly acted as tumor suppressors [50–52]. However, further investigation showed that only miR-218-5p upregulation significantly repressed LASP1 mRNA expression in BxPC-3 cells (Additional file 6: Figure S2D). Moreover, miR-218 was downregulated in pancreatic cancer, and reduced miR-218 was associated with poor prognosis of pancreatic cancer patients [29]. MiR-218 upregulation inhibited the proliferation and invasion and induced apoptosis of pancreatic cancer cells [30]. In the present study, we showed that miR-218-5p upregulation repressed pancreatic cancer cell growth, migration and invasion by directly regulating LASP1 (Fig. 5). In line with our results, miR-218-5p upregulation inhibited prostate cancer cell migration and invasion by directly regulating LASP1 expression [32]. Moreover, miR-218-5p downregulation partially reversed the inhibition of LASP1 expression, cell proliferation, colony formation, cell migration and cell invasion caused by ANLN knockdown (Fig. 6). These results suggested that ANLN induces the expression of LASP1 by repressing the expression of miR-218-5p, resulting in pancreatic cancer cell progression.

Additionally, EZH2-mediated H3K27 trimethylation is involved in epigenetic silencing of miR-218 [37, 53]. For example, EZH2 induces histone methylation and heterochromatin formation at miR-218-2 promoter that leads to miR-218 downregulation in pancreatic cancer, thereby mediating cell proliferation, cell migration and cell invasion in vitro and tumor growth and metastasis in vivo [37]. Interestingly, our results showed that ANLN knockdown significantly inhibited the expression of EZH2 in (Additional file 7: Figure S3). Moreover, miR-218-5p downregulation partially reversed the inhibition of LASP1 expression induced by EZH2 knockdown (Fig. 7c). Thus, we hypothesized that ANLN may induce the silencing of miR-218-5p by mediating EZH2 in pancreatic cancer progression. Our results support this, as we found that EZH2 restoration obviously reversed the upregulation of miR-218-5p and the inhibition of LASP1 expression, cell proliferation, colony formation, cell migration and cell invasion caused by ANLN knockdown (Fig. 7d-h). We further demonstrated that ANLN knockdown significantly inhibited the levels of EZH2 and LASP1 expression in xenograft tumor models (Fig. 8). Taken together, EZH2 induced by ANLN may promote pancreatic cancer progression by regulating miR-218-5p/LASP1 signaling axis (Fig. 9).

Although previous reports and our results suggest that LASP1 contributes to pancreatic cancer cell growth and metastasis, the downstream mechanisms of LASP1 remains unclear in pancreatic cancer. Zhou R et al. reported that COPS5 and LASP1 synergistically interacted to induce colorectal cancer progression by PI3K/AKT pathway [20]. In non-small cell lung cancer (NSCLC), LASP1 directly bound to FAK and enhanced the phosphorylation of FAK and AKT, thereby inducing cell proliferation and invasion [54]. Gao QZ et al. found that LASP1 regulated nasopharyngeal carcinoma cell aggressiveness via LASP1/PTEN/PI3K/AKT axis [55]. In addition, PI3K/AKT axis is frequently activated in pancreatic cancer and is essential for pancreatic cancer progression [56]. Therefore, vigorous research efforts are needed to further clarify whether PI3K/AKT axis contributed to LASP1-mediated pancreatic cancer cell growth and metastasis. Interestingly, previous study reported that PI3K/AKT signaling promoted the malignant potential of lung cancer cells by regulating ANLN nuclear localization and stability [17]. Thus, the role and mechanisms of PI3K/AKT signaling in regulating ANLN remained to be further elucidated.

## Conclusions

In summary, we have identified the ANLN/EZH2/miR-218-5p/LASP1 signaling axis in pancreatic cancer cells, which provides new insights into the mechanism underlying pancreatic cancer progression and generates a mechanism-based novel framework for developing therapeutics in pancreatic cancer treatment.

## Additional files

Additional file 1: **Table S1.** siRNAs, shRNA and primers. (DOCX 20 kb)

Additional file 2: **Table S2.** GO terms representing biological process. (DOCX 17 kb)

Additional file 3: **Table S3.** GO terms representing molecular function. (DOCX 17 kb)

Additional file 4: **Table S4.** GO terms representing cellular compartment. (DOCX 19 kb)

Additional file 5: **Figure S1.** ANLN knockdown inhibited BxPC-3 cell proliferation by mediating LASP1. (A) Based on the GENT database, LASP1, RAB11B, RUVBL1 and MYO1B gene expression were analyzed in the pancreatic cancer tissues and normal pancreatic tissues. (B) There were significant Pearson correlations of ANLN with LASP1 and ANLN with RUVBL1 in the pancreatic cancer tissues and the normal pancreatic tissues. (C) CCK-8 assay showed that LASP1 restoration partially reversed the effects of ANLN knockdown on pancreatic cancer cell proliferation, while RUVBL1 restoration did not reverse the effect of ANLN downregulation on pancreatic cancer cell proliferation. **P < 0.01. (JPG 1806 kb)

Additional file 6: **Figure S2.** MiR-218-5p upregulation significantly repressed the expression of LASP1 mRNA in BxPC-3. (A) The heat map of the differentially expressed miRNA precursors with a fold change of greater than 2 or less than − 2 in ANLN RNAi relative to NC. (B) The selected candidate miRNAs (miR-145-5p, miR-218-5p and miR-9-5p) were confirmed by qRT-PCR after ANLN RNAi transfection in BxPC-3 cells. (C) The expression levels of miR-145-5p, miR-218-5p and miR-9-5p were detected by qRT-PCR in BxPC-3 cells transfected with the miR-145-5p mimic (miR-145-5p), miR-218-5p mimic (miR-218-5p), miR-9-5p mimic (miR-9-5p) or mimic control (con). U6 was used as a loading control. (D) The expression levels of LASP1 mRNA in BxPC-3 cells transfected with the miR-145-5p mimic (miR-145-5p), miR-218-5p mimic (miR-218-5p), miR-9-5p mimic (miR-9-5p) or mimic control (con) were analyzed by qRT-PCR. **P < 0.01. (JPG 1590 kb)

Additional file 7: **Figure S3.** ANLN depletion significantly repressed the expression of EZH2 mRNA and protein in BxPC-3 and SW1990 cells. (A) The heat map of the selected genes. (B and C) The expression of EZH2 mRNA and protein was determined by qRT-PCR and Western blot in BxPC-3 and SW1990 cells transfected with the ANLN siRNA (ANLN RNAi) or the scramble control (NC). (JPG 512 kb)

## Data Availability

All data generated or analyzed during this study are included in this published article and its additional files.

## Abbreviations

ANLN: Anillin actin binding protein
CCK-8: Cell counting kit-8
EZH2: Enhancer of zeste homolog 2
GO: Gene ontology
IHC: Immunohistochemistry
LASP1: LIM and SH3 protein 1
MOI: Multiplicity of infection
OS: Overall survival
qRT-PCR: Quantitative reverse transcription polymerase chain reaction
shRNA: Short hairpin RNA
siRNA: Small interfering RNA
UTR: Untranslated region
