# Supplementary material for: ANLN-induced EZH2 upregulation promotes pancreatic cancer progression by mediating miR-218-5p/LASP1 signaling axis
Source: J Exp Clin Cancer Res. 2019 Aug 8;38:347. doi: 10.1186/s13046-019-1340-7 (PMC6686567; doi:10.1186/s13046-019-1340-7)
Supplement: Supplementary file 1 — Table S1. siRNAs, shRNA and primers. (DOCX 20 kb) [file 13046_2019_1340_MOESM1_ESM.docx]

**Table S1.** siRNAs, shRNA and primers

| Name | Sequence |
| --- | --- |
| ANLN siRNA | 5’-GCAAACAACUAGAAACCAATT-3’ |
| NC | 5’-UUCUCCGAACGUGUCACGUTT-3’ |
| EZH2 siRNA | 5’- GGGAUAGAGAAUGUGGGUUUAUAAATT-3’ |
| LV-ANLN shRNA | 5’-CCGGGCAAACAACTAGAAACCAACTCGAGTTGGTTTCTAGTTGTTTGC TTTTTTG-3’ |
| LV-NC | 5’-CCGGTTCTCCGAACGTGTCACGTTTCAAGAGAACGTGACACGTTCGGAGAATTTTTG-3’ |
| ANLN Fwd | 5’-CAAGATGTATCCAATGACT-3’ |
| ANLN Rev | 5’-TGACTGAAGAATGAATGTT-3’ |
| EZH2 Fwd | 5’-AGGACGGCTCCTCTAACCAT-3’ |
| EZH2 Rev | 5’-CTTGGTGTTGCACTGTGCTT-3’ |
| β-actin Fwd | 5’-CATGTACGTTGCTATCCAGGC -3’ |
| β-actin Rev | 5’-CTCCTTAATGTCACGCACGAT -3’ |
| LASP1 Fwd | 5’- GAAGAAGCCCTACTGCAACG-3’ |
| LASP1 Rev | 5’- TGATCTGGTCCTGGGTCTTC-3’ |
| RPS26 Fwd | 5’-GAACGCATTTCCACCCTAGA-3’ |
| RPS26 Rev | 5’-GCACGACCATTGTTCCTTCT-3’ |
| EIF2S3 Fwd | 5’-CTGTCCTGGCCACGATATTT-3’ |
| EIF2S3 Rev | 5’-CCTTGGACAAATGCAAGGAT-3’ |
| RPL7A Fwd | 5’-AAAATGGGGGTCCCTTACTG-3’ |
| RPL7A Rev | 5’-CTTTTCGAGCTTGGCGATAC-3’ |
| RAB11B Fwd | 5’-GCAGACAGCAACATCGTCAT-3’ |
| RAB11B Rev | 5’-TGTGACACGATGCGGTAGAT-3’ |
| HIST1H3B Fwd | 5’-AGTCGACCGAGTTGCTGATT-3’ |
| HIST1H3B Rev | 5’-TCCTCAAAGAGCCCTACCAA-3’ |
| RUVBL1 Fwd | 5’-GCCAGCTAATGAAGCCAAAG-3’ |
| RUVBL1 Rev | 5’-GAAGCACTCAATGTCCAGCA-3’ |
| HIST1H3I Fwd | 5’-AAAGCAAACAGCTCGCAAGT-3’ |
| HIST1H3I Rev | 5’-GCTCGGTCGACTTCTGGTAG-3’ |
| RANBP1 Fwd | 5’-GTGCCAACCACTACATCACG-3’ |
| RANBP1 Rev | 5’-TGCCTGATCCTGCTTTCTTT-3’ |
| MYO1B Fwd | 5’-AACATGGCCTCATTGGAAAG-3’ |
| MYO1B Rev | 5’-CGTTGCTTCCTCAGGTCTTC-3’ |
| NOP56 Fwd | 5’-TGGCTCCAAGAAAAAGAGGA-3’ |
| NOP56 Rev | 5’-GGAAATGTCACCTTGGGCTA-3’ |
| miR-218-5p RT | 5’-GTCGTATCCAGTGCAGGGTCCGAGGTGCACTGGATACGACACATGGT-3’ |
| miR-218-5p Fwd | 5’-GGGGGTTGTGCTTGATCTAAC-3’ |
| miR-145-5p RT | 5’-GTCGTATCCAGTGCAGGGTCCGAGGTGCACTGGATACGACAGGGATT-3’ |
| miR-145-5p Fwd | 5’-GGTCCAGTTTTCCCAGGA-3’ |
| miR-9-5p RT | 5’- GTCGTATCCAGTGCAGGGTCCGAGGTGCACTGGATACGACTCATACA -3’ |
| miR-9-5p Fwd | 5’-GGGGGTCTTTGGTTATCTAGCTG-3’ |
| U6 RT | 5’-GTCGTATCCAGTGCAGGGTCCGAGGTGCACTGGATACGACAAAATATGGAAC-3’ |
| U6 Fwd | 5’-GTGCTCGCTTCGGCAGC-3’ |
| Rev | 5’-CAGTGCAGGGTCCGAGGT-3’ |
