# Supplementary material for: ANLN-induced EZH2 upregulation promotes pancreatic cancer progression by mediating miR-218-5p/LASP1 signaling axis
Source: J Exp Clin Cancer Res. 2019 Aug 8;38:347. doi: 10.1186/s13046-019-1340-7 (PMC6686567; doi:10.1186/s13046-019-1340-7)
Supplement: Supplementary file 2 — Table S2. GO terms representing biological process. (DOCX 17 kb) [file 13046_2019_1340_MOESM2_ESM.docx]

**Table S2. GO terms representing biological process**

| GO ID | Biological process (GO description) | *P*-value | Count |
| --- | --- | --- | --- |
| GO: 0006364 | rRNA processing | 2.6596E-11 | 63 |
| GO: 0031047 | gene silencing by RNA | 2.7594E-10 | 40 |
| GO: 0006409 | tRNA export from nucleus | 1.8798E-7 | 17 |
| GO: 0045815 | positive regulation of gene expression, epigenetic | 4.0410E-7 | 24 |
| GO: 0006406 | mRNA export from nucleus | 4.6659E-7 | 32 |
| GO: 0007077 | mitotic nuclear envelope disassembly | 1.3878E-6 | 19 |
| GO: 0006260 | DNA replication | 2.3362E-6 | 41 |
| GO: 0006270 | DNA replication initiation | 8.1225E-6 | 15 |
| GO: 0006412 | translation | 1.084E-5 | 56 |
| GO: 0010827 | regulation of glucose transport | 1.2534E-5 | 15 |
| GO: 0016925 | protein sumoylation | 1.756E-5 | 32 |
| GO: 0006413 | translational initiation | 3.14789E-5 | 35 |
| GO: 0006335 | DNA replication-dependent nucleosome assembly | 4.4601E-5 | 14 |
| GO: 0000183 | chromatin silencing at rDNA | 5.8309E-5 | 15 |
| GO: 0000082 | G1/S transition of mitotic cell cycle | 6.0800E-5 | 28 |
| GO: 0051437 | positive regulation of ubiquitin-protein ligase activity involved in regulation of mitotic cell cycle transition | 6.7510E-5 | 23 |
| GO: 0051290 | protein heterotetramerization | 6.9845E-5 | 16 |
| GO: 0007067 | mitotic nuclear division | 1.0263E-4 | 52 |
| GO:0006904 | vesicle docking involved in exocytosis | 1.1148E-4 | 12 |
| GO: 0031145 | anaphase-promoting complex-dependent catabolic process | 1.2718E-4 | 23 |
| GO: 0006334 | nucleosome assembly | 1.6427E-4 | 30 |
| GO: 0045814 | negative regulation of gene expression, epigenetic | 1.8061E-4 | 17 |
| GO: 0051436 | negative regulation of ubiquitin-protein ligase activity involved in mitotic cell cycle | 2.1425E-4 | 21 |
| GO: 0098609 | cell-cell adhesion | 2.8715E-4 | 54 |
| GO: 0006268 | DNA unwinding involved in DNA replication | 4.3331E-4 | 7 |
