# Supplementary material for: ANLN-induced EZH2 upregulation promotes pancreatic cancer progression by mediating miR-218-5p/LASP1 signaling axis
Source: J Exp Clin Cancer Res. 2019 Aug 8;38:347. doi: 10.1186/s13046-019-1340-7 (PMC6686567; doi:10.1186/s13046-019-1340-7)
Supplement: Supplementary file 3 — Table S3. GO terms representing molecular function. (DOCX 17 kb) [file 13046_2019_1340_MOESM3_ESM.docx]

**Table S3. GO terms representing molecular function**

| GO ID | Molecular function (GO description) | *P*-value | Count |
| --- | --- | --- | --- |
| GO: 0044822 | poly(A) RNA binding | 7.2539E-23 | 249 |
| GO:0005515 | protein binding | 1.5825E-16 | 1231 |
| GO:0098641 | cadherin binding involved in cell-cell adhesion | 6.2572E-5 | 59 |
| GO:0031492 | nucleosomal DNA binding | 2.0381E-4 | 16 |
| GO:0004004 | ATP-dependent RNA helicase activity | 4.889E-4 | 19 |
| GO:0003735 | structural constituent of ribosome | 0.0010 | 44 |
| GO:0008139 | nuclear localization sequence binding | 0.0011 | 11 |
| GO:0003678 | DNA helicase activity | 0.0013 | 10 |
| GO:0019899 | enzyme binding | 0.0014 | 60 |
| GO:0000166 | nucleotide binding | 0.0015 | 62 |
| GO:0003743 | translation initiation factor activity | 0.0018 | 17 |
| GO:0008536 | Ran GTPase binding | 0.0019 | 11 |
| GO:0005524 | ATP binding | 0.0023 | 216 |
| GO:0042393 | histone binding | 0.0025 | 27 |
| GO:0003723 | RNA binding | 0.0026 | 89 |
| GO:0003729 | mRNA binding | 0.0035 | 27 |
| GO:0003899 | DNA-directed RNA polymerase activity | 0.0041 | 12 |
| GO:0001104 | RNA polymerase II transcription cofactor activity | 0.0083 | 11 |
| GO:0008327 | methyl-CpG binding | 0.0091 | 8 |
| GO:0004298 | threonine-type endopeptidase activity | 0.0091 | 8 |
| 0017056 | structural constituent of nuclear pore | 0.0091 | 8 |
| GO:0015450 | P-P-bond-hydrolysis-driven protein transmembrane transporter activity | 0.0098 | 5 |
| GO:0008137 | NADH dehydrogenase (ubiquinone) activity | 0.0099 | 13 |
| GO:0005487 | nucleocytoplasmic transporter activity | 0.0120 | 8 |
| GO:0003924 | GTPase activity | 0.0138 | 41 |
