# Supplementary material for: ANLN-induced EZH2 upregulation promotes pancreatic cancer progression by mediating miR-218-5p/LASP1 signaling axis
Source: J Exp Clin Cancer Res. 2019 Aug 8;38:347. doi: 10.1186/s13046-019-1340-7 (PMC6686567; doi:10.1186/s13046-019-1340-7)
Supplement: Supplementary file 4 — Table S4. GO terms representing cellular compartment. (DOCX 19 kb) [file 13046_2019_1340_MOESM4_ESM.docx]

**Table S4. GO terms representing cellular compartment**

| GO ID | Cellular component (GO description) | *P*-value | Count |
| --- | --- | --- | --- |
| GO: 0005654 | nucleoplasm | 1.5773E-31 | 525 |
| GO:0016020 | membrane | 3.6009E-16 | 384 |
| GO:0005739 | mitochondrion | 1.3489E-11 | 240 |
| GO:0005730 | nucleolus | 2.0884E-11 | 169 |
| GO:0005829 | cytosol | 1.5989E-10 | 505 |
| GO:0000786 | nucleosome | 2.2670E-7 | 31 |
| GO:0005643 | nuclear pore | 3.7750E-7 | 26 |
| GO:0005743 | mitochondrial inner membrane | 1.2053E-6 | 88 |
| GO:0000784 | nuclear chromosome, telomeric region | 2.2118E-6 | 36 |
| GO:0000228 | nuclear chromosome | 2.2458E-5 | 19 |
| GO:0005737 | cytoplasm | 3.0849E-5 | 703 |
| GO:0005765 | lysosomal membrane | 3.2913E-5 | 57 |
| GO:0005759 | mitochondrial matrix | 3.8272E-5 | 65 |
| GO:0005913 | cell-cell adherens junction | 4.8764E-5 | 64 |
| GO:0005635 | nuclear envelope | 9.6498E-5 | 37 |
| GO:0005789 | endoplasmic reticulum membrane | 1.010E-4 | 140 |
| GO:0070062 | extracellular exosome | 2.482E-4 | 392 |
| GO:0016605 | PML body | 3.9869E-4 | 25 |
| GO:0031965 | nuclear membrane | 4.6380E-4 | 46 |
| GO:0000940 | condensed chromosome outer kinetochore | 9.7533E-4 | 6 |
| GO:0042555 | MCM complex | 0.0020 | 6 |
| GO:0005840 | ribosome | 0.0020 | 34 |
| GO:0030176 | integral component of endoplasmic reticulum membrane | 0.0023 | 24 |
| GO:0005762 | mitochondrial large ribosomal subunit | 0.0032 | 14 |
| GO:0043231 | intracellular membrane-bounded organelle | 0.0034 | 89 |
