# Supplementary figures and images for: ANLN-induced EZH2 upregulation promotes pancreatic cancer progression by mediating miR-218-5p/LASP1 signaling axis
Source: J Exp Clin Cancer Res. 2019 Aug 8;38:347. doi: 10.1186/s13046-019-1340-7 (PMC6686567; doi:10.1186/s13046-019-1340-7)

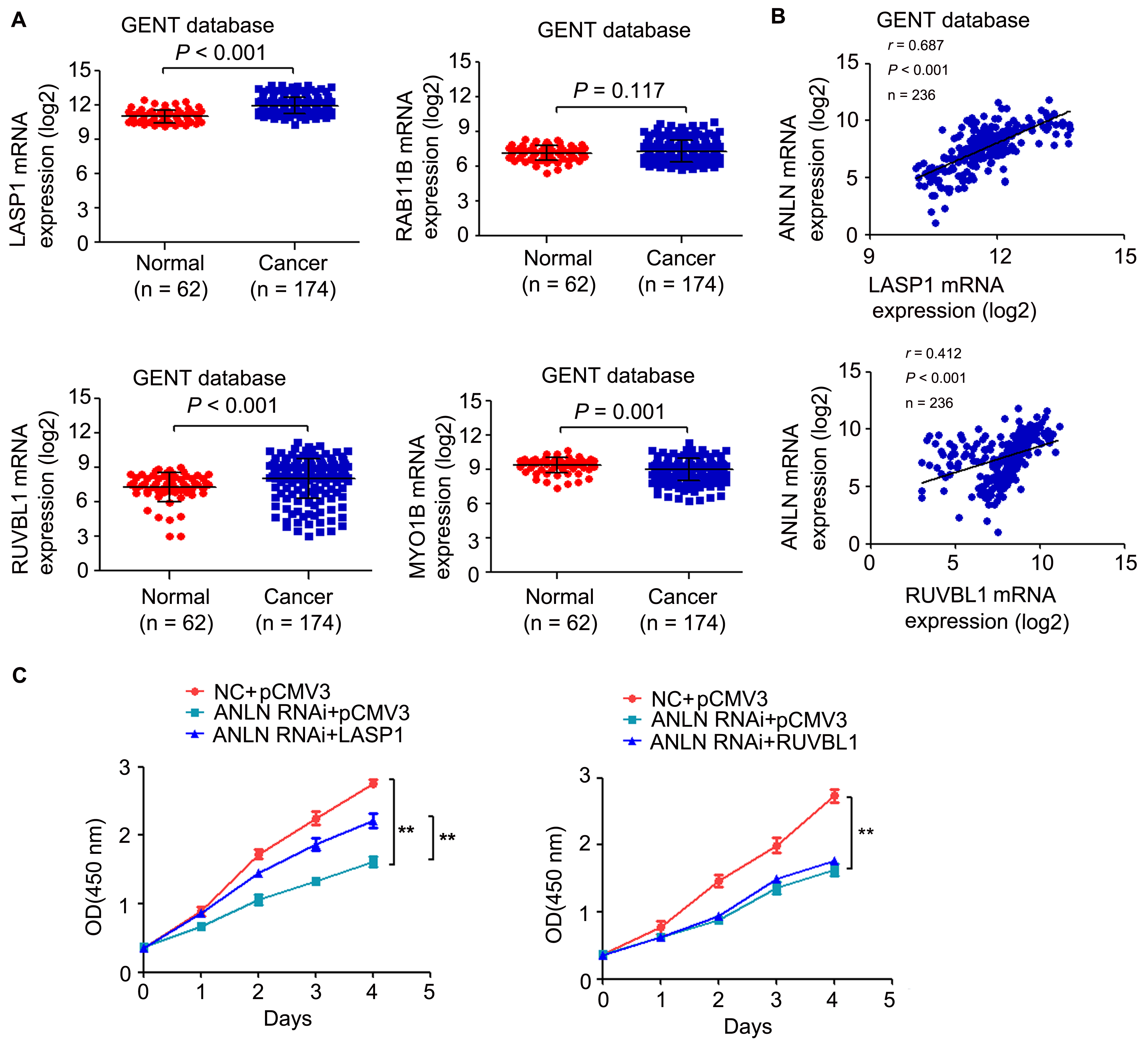

Supplement: Supplementary file 5 — Figure S1. ANLN knockdown inhibited BxPC-3 cell proliferation by mediating LASP1. (A) Based on the GENT database, LASP1, RAB11B, RUVBL1 and MYO1B gene expression were analyzed in the pancreatic cancer tissues and normal pancreatic tissues. (B) There were significant Pearson correlations of ANLN with LASP1 and ANLN with RUVBL1 in the pancreatic cancer tissues and the normal pancreatic tissues. (C) CCK-8 assay showed that LASP1 restoration partially reversed the effects of ANLN knockdown on pancreatic cancer cell proliferation, while RUVBL1 restoration did not reverse the effect of ANLN downregulation on pancreatic cancer cell proliferation. **P < 0.01. (JPG 1806 kb) [file 13046_2019_1340_MOESM5_ESM.jpg]

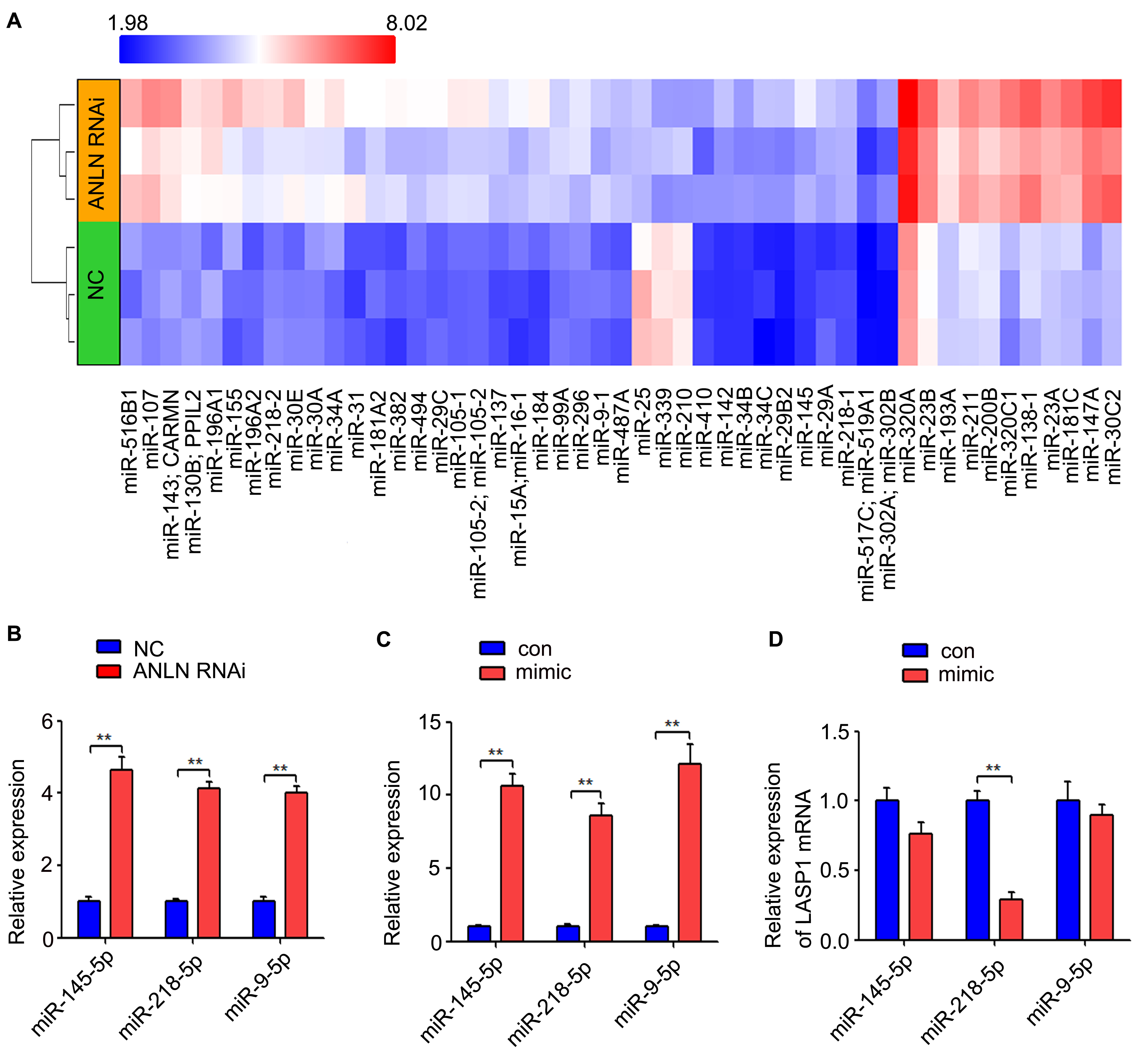

Supplement: Supplementary file 6 — Figure S2. MiR-218-5p upregulation significantly repressed the expression of LASP1 mRNA in BxPC-3. (A) The heat map of the differentially expressed miRNA precursors with a fold change of greater than 2 or less than − 2 in ANLN RNAi relative to NC. (B) The selected candidate miRNAs (miR-145-5p, miR-218-5p and miR-9-5p) were confirmed by qRT-PCR after ANLN RNAi transfection in BxPC-3 cells. (C) The expression levels of miR-145-5p, miR-218-5p and miR-9-5p were detected by qRT-PCR in BxPC-3 cells transfected with the miR-145-5p mimic (miR-145-5p), miR-218-5p mimic (miR-218-5p), miR-9-5p mimic (miR-9-5p) or mimic control (con). U6 was used as a loading control. (D) The expression levels of LASP1 mRNA in BxPC-3 cells transfected with the miR-145-5p mimic (miR-145-5p), miR-218-5p mimic (miR-218-5p), miR-9-5p mimic (miR-9-5p) or mimic control (con) were analyzed by qRT-PCR. **P < 0.01. (JPG 1590 kb) [file 13046_2019_1340_MOESM6_ESM.jpg]

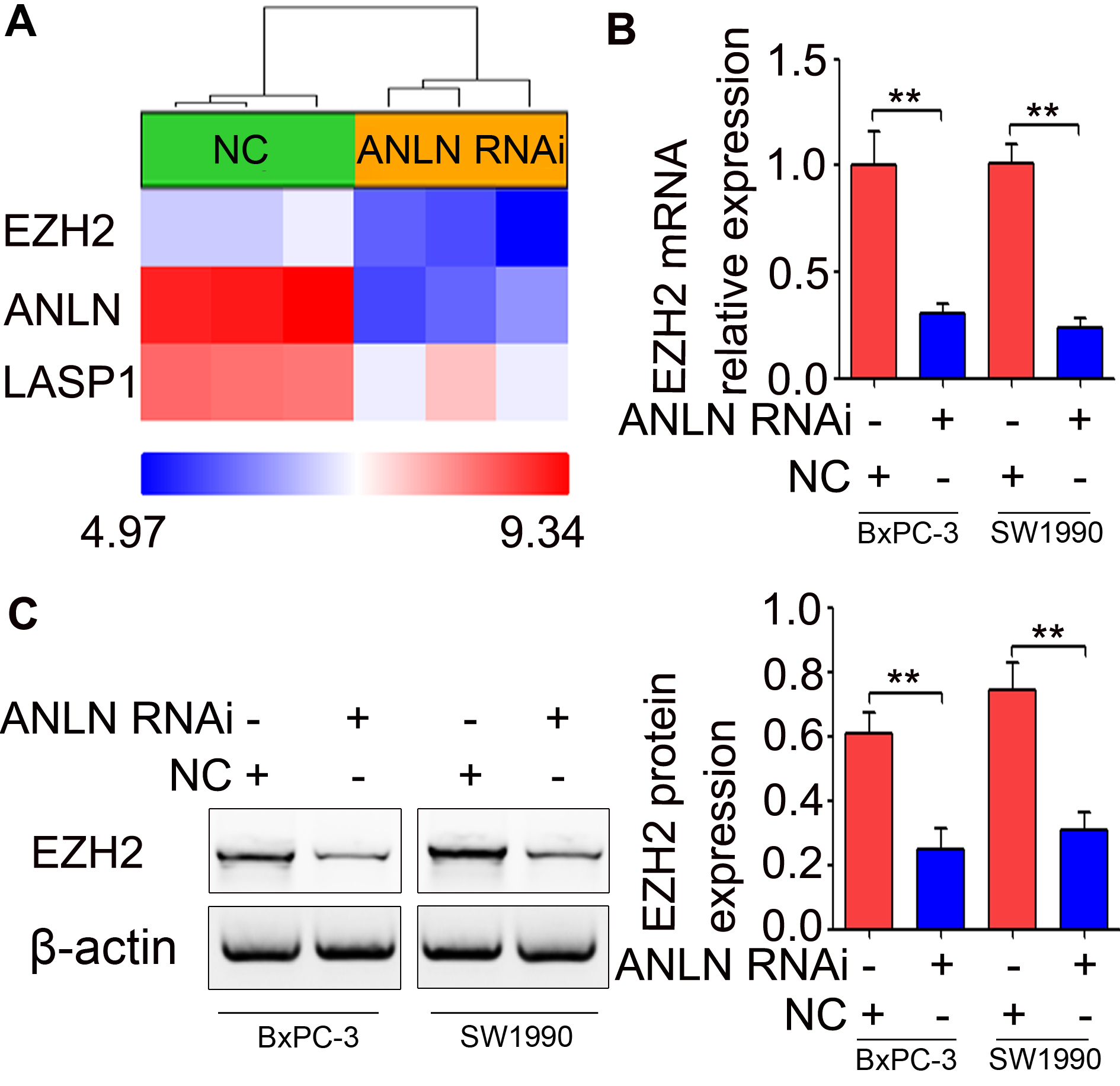

Supplement: Supplementary file 7 — Figure S3. ANLN depletion significantly repressed the expression of EZH2 mRNA and protein in BxPC-3 and SW1990 cells. (A) The heat map of the selected genes. (B and C) The expression of EZH2 mRNA and protein was determined by qRT-PCR and Western blot in BxPC-3 and SW1990 cells transfected with the ANLN siRNA (ANLN RNAi) or the scramble control (NC). (JPG 512 kb) [file 13046_2019_1340_MOESM7_ESM.jpg]
